# The role of chloroplast movement in C_4_ photosynthesis: a theoretical analysis using a three-dimensional reaction–diffusion model for maize

**DOI:** 10.1093/jxb/erad138

**Published:** 2023-04-21

**Authors:** Moges A Retta, Xinyou Yin, Quang Tri Ho, Rodrigo Watté, Herman N C Berghuijs, Pieter Verboven, Wouter Saeys, Francisco Javier Cano, Oula Ghannoum, Paul C Struik, Bart M Nicolaï

**Affiliations:** KU Leuven, MeBioS division, Willem de Croylaan 42, B-3001, Leuven, Belgium; Centre for Crop Systems Analysis, Wageningen University & Research, P.O. Box 430, 6700 AK Wageningen, The Netherlands; Centre for Crop Systems Analysis, Wageningen University & Research, P.O. Box 430, 6700 AK Wageningen, The Netherlands; Institute of Marine Research, Nordnesgaten 50, NO-5005 Bergen, P.O. Box 1870, Nordnes, Norway; KU Leuven, MeBioS division, Willem de Croylaan 42, B-3001, Leuven, Belgium; Plant Production Systems group, Wageningen University & Research, P.O. Box 430, 6700 AK Wageningen, The Netherlands; KU Leuven, MeBioS division, Willem de Croylaan 42, B-3001, Leuven, Belgium; KU Leuven, MeBioS division, Willem de Croylaan 42, B-3001, Leuven, Belgium; Centro de Investigación Forestal (CIFOR), Instituto Nacional de Investigacion y Tecnologia Agraria y Alimentaria (INIA), Consejo Superior de Investigaciones Científicas (CSIC), Carretera de la Coruña Km 7.5, 28040, Madrid, Spain; ARC Centre of Excellence for Translational Photosynthesis, Hawkesbury Institute for the Environment, University of Western Sydney, Hawkesbury campus, Locked Bag 1797, Penrith 2751, NSW, Australia; ARC Centre of Excellence for Translational Photosynthesis, Hawkesbury Institute for the Environment, University of Western Sydney, Hawkesbury campus, Locked Bag 1797, Penrith 2751, NSW, Australia; Centre for Crop Systems Analysis, Wageningen University & Research, P.O. Box 430, 6700 AK Wageningen, The Netherlands; KU Leuven, MeBioS division, Willem de Croylaan 42, B-3001, Leuven, Belgium; Flanders Center of Postharvest Technology, Willem de Croylaan 42, B-3001, Leuven, Belgium; Lancaster University, UK

**Keywords:** Biophysical model, chloroplast movement, CO_2_ concentrating mechanism, gas exchange, leakiness, ray tracing, 3D leaf anatomy

## Abstract

Chloroplasts movement within mesophyll cells in C_4_ plants is hypothesized to enhance the CO_2_ concentrating mechanism, but this is difficult to verify experimentally. A three-dimensional (3D) leaf model can help analyse how chloroplast movement influences the operation of the CO_2_ concentrating mechanism. The first volumetric reaction–diffusion model of C_4_ photosynthesis that incorporates detailed 3D leaf anatomy, light propagation, ATP and NADPH production, and CO_2_, O_2_ and bicarbonate concentration driven by diffusional and assimilation/emission processes was developed. It was implemented for maize leaves to simulate various chloroplast movement scenarios within mesophyll cells: the movement of all mesophyll chloroplasts towards bundle sheath cells (aggregative movement) and movement of only those of interveinal mesophyll cells towards bundle sheath cells (avoidance movement). Light absorbed by bundle sheath chloroplasts relative to mesophyll chloroplasts increased in both cases. Avoidance movement decreased light absorption by mesophyll chloroplasts considerably. Consequently, total ATP and NADPH production and net photosynthetic rate increased for aggregative movement and decreased for avoidance movement compared with the default case of no chloroplast movement at high light intensities. Leakiness increased in both chloroplast movement scenarios due to the imbalance in energy production and demand in mesophyll and bundle sheath cells. These results suggest the need to design strategies for coordinated increases in electron transport and Rubisco activities for an efficient CO_2_ concentrating mechanism at very high light intensities.

## Teaser

A novel 3D reaction–diffusion model of gas transport during C_4_photosynthesis revealed that the movement of mesophyll chloroplasts increased rate of photosynthesis at the cost of increased leakiness.

## Introduction

Photosynthesis in C_4_ plants is empowered by the CO_2_ concentrating mechanism (CCM) that enables a higher CO_2_ concentration in bundle sheath chloroplasts, allowing ribulose-1,5-bisphosphate carboxylase/oxygenase (Rubisco) to operate near a CO_2_ saturation level. During C_4_ photosynthesis, the CO_2_ that diffuses from the intercellular air spaces (IAS) to the mesophyll cytosol is hydrated to bicarbonate, which reacts with phosphoenolpyruvate (PEP) in the presence of phosphoenolpyruvate carboxylase (PEPC) to form C_4_ acids. The C_4_ acids diffuse to bundle sheath cells and are decarboxylated to release CO_2_ for fixation by Rubisco in bundle sheath chloroplasts (Furbank and Hatch, 1987; Furbank, 2011). The affinity of PEPC for bicarbonate is much higher than that of Rubisco for CO_2_, and the PEP carboxylation is much faster than RuBP carboxylation by Rubisco. This increases the CO_2_ concentration in bundle sheath chloroplasts, which supercharges the catalytic sites of Rubisco, reduces photorespiration, and thus enhances the photosynthetic rate (Kanai and Edwards, 1999). An efficient operation of the CCM requires the coordinated functioning of mesophyll and bundle sheath cells (Pengelly *et al.*, 2010; Kromdijk *et al.*, 2010, 2014; Sun *et al.*, 2012, 2014; Bellasio and Lundgren, 2016) as the CCM is more costly in ATP to regenerate PEP in mesophyll cells. The concentric arrangement of mesophyll, bundle sheath, and vasculature, the relative size of mesophyll and bundle sheath cells, and chloroplast abundance and distribution impact the light harvesting potential of bundle sheath cells (Evans *et al.*, 2007; Stata *et al.*, 2014; Bellasio and Lundgren, 2016) influencing the operation of the CCM.

Mesophyll chloroplasts of C_4_ plants are randomly distributed along the cell walls while those of bundle sheath cells are typically close to mesophyll cells (centrifugal location), which is an arrangement common in the classical NADP-ME subtype of C_4_ plants that includes major C_4_ crops (Dengler and Nelson, 1999). Upon exposure to high light for several hours or at extremely high light intensity at midday, mesophyll chloroplasts of finger millet (*Eleusine coracana*, a NAD-ME species) exhibited movement towards bundle sheath cells (referred to as aggregative movement) (Maai *et al.*, 2020a). Under similar conditions, mesophyll chloroplast of NADP-ME species, like sorghum and maize, aligned between veins, along the sides of anticlinal walls, and parallel to the direction of light to allow light to pass through (referred to as avoidance movement) (Yamada *et al.*, 2009; Maai *et al.*, 2020b). When exposed to blue light after dark adaptation for several hours, mesophyll chloroplasts of maize and sorghum showed aggregative movement (Ryu *et al.*, 2014). In addition, the aggregative movement has recently been shown to occur in a number of other C_4_ plants (Kato *et al.*, 2022). Unlike mesophyll chloroplasts, the intracellular arrangement of bundle sheath chloroplasts was unaffected in finger millet, maize, or sorghum (Taniguchi *et al.*, 2003; Yamada *et al.*, 2009; Maai *et al.*, 2020a).

C_4_ crops experience high light for a prolonged time in their natural environment, especially at the top of the canopy, which may compromise yield (Anderson *et al.*, 2021; Wang *et al.*, 2022). Given the variety of arrangements of mesophyll chloroplasts in response to high light intensity, it is important to understand what the consequences of each chloroplast configuration are for the leaf energetics and net photosynthetic rate (*A*_n_). The analysis requires knowing the amount of light absorbed by mesophyll and bundle sheath chloroplasts to analyse the cell-specific ATP and NADPH demand and production, which are hard to measure but important parameters of C_4_ energetics (Bellasio and Lundgren, 2016; Yin and Struik, 2018). The CCM efficiency is quantified by leakiness (Φ), defined as the ratio of CO_2_ leakage flux from bundle sheath cells to the rate of PEP carboxylation (Kanai and Edwards, 1999; Yin and Struik, 2018).

Chloroplast movement in C_3_ plants in response to light and its effect on CO_2_ assimilation and productivity have been extensively investigated (Tholen *et al.*, 2008; Wada, 2013; Gotoh *et al.*, 2018). In C_4_ plants, chloroplast movement following short term changes in light quality in maize was proposed to result in better coordination between CO_2_ fixation in mesophyll and bundle sheath cells, and thus reduced Φ (Sun *et al.*, 2014). The aggregative movement is assumed to boost photosynthesis through improved light penetration or by shortening the diffusion path of metabolites and re-fixation of leakage CO_2_ (Taniguchi *et al.*, 2003; Yamada *et al.*, 2009; Maai *et al.*, 2011, 2020a; Taniguchi and Cousins, 2018). However, it was found that the extent of aggregative movement did not differ for different bundle sheath chloroplast positions and for the presence or absence of a suberin lamella in bundle sheath cells, factors that may affect CO_2_ leakage, its refixation and metabolite transport (Kato *et al.*, 2022). By contrast, the avoidance movement, which is associated with photoprotection of mesophyll chloroplasts in sorghum, may decrease *A*_n_ (Maai *et al.*, 2020b).

The effect of chloroplast movement on the energetics of C_4_ plants and photosynthetic rate can be analysed in a controlled manner using modeling due to otherwise simultaneous changes in leaf biochemistry, stomatal conductance, and anatomy of C_4_ plants upon exposure to high light stress (Maai *et al.*, 2020b; Anderson *et al.*, 2021). C_4_ leaf energetics were recently examined using biochemical models and flux balance analysis (Bellasio and Lundgren, 2016; Bellasio, 2017; Yin and Struik, 2018, 2021; Bellasio and Farquhar, 2019). Some of these studies (Bellasio and Lundgren, 2016; Yin and Struik, 2018) accounted for the effect of leaf anatomy on light propagation indirectly using the Beer–Lambert law (Bellasio and Lundgren, 2016; Yin and Struik, 2018). The light propagation was modeled using the meshed Monte Carlo method, which, unlike the classical Monte Carlo method for photon transport, uses tetrahedral meshes for better representation of the structural intricacies of biological tissues (Watté *et al.*, 2015; Ho *et al.*, 2016). Reaction–diffusion (R-D) models of CO_2_ transport account for the effect of leaf anatomy on light and CO_2_ distribution directly (Berghuijs *et al.*, 2016). R-D models were applied to analyse gas exchange during photosynthesis using two-dimensional (2D) (Ho *et al.*, 2012; Retta *et al.*, 2016a; Berghuijs *et al.*, 2019) or three-dimensional (3D) (Tholen and Zhu, 2011; Ho *et al.*, 2016; Wang *et al*., 2017; Xiao *et al.*, 2023) leaf anatomy. However, 3D R-D models provide the capacity for more accurate and mechanistic analysis of CO_2_ and light propagation as emphasized recently (Earles *et al.*, 2019). Exploring the influence of a 3D arrangement of mesophyll and bundle sheath chloroplasts on photosynthesis requires a realistic 3D geometry at tissue level for accurate modeling of light propagation and diffusion of gases for the irregular geometry of the IASs (Ho *et al.*, 2016; Xiao *et al.*, 2016; Théroux-Rancourt *et al.*, 2017; Earles *et al.*, 2019). Yet the application of such models to C_4_ photosynthesis has received less attention than for C_3_ plants.

To quantify the effect of chloroplast movement on C_4_ energetics, photosynthesis, and CCM efficiency, we develop a 3D model of C_4_ photosynthesis consisting of a R-D model of gas exchange coupled with an ATP and NADPH production model (Yin and Struik, 2018) and a light propagation model (Watté *et al.*, 2015) using a 3D representation of a real maize (*Zea mays* L.) leaf as a model system. We hypothesized that the aggregative movement of mesophyll chloroplasts improves photosynthesis through improved light propagation, increased energy production, and reduced leakiness. The avoidance movement of mesophyll chloroplasts is hypothesized to decrease photosynthetic rate at high light intensity.

## Materials and methods

### Plant sample, experiments, and reconstruction of the 3D leaf anatomy

The plant growth conditions, gas exchange, and chlorophyll fluorescence measurements were described in detail previously (Retta *et al.*, 2016a). In brief, maize plants (hybrid 2-02R10074) were grown receiving contrasting nitrogen treatments. The growth conditions were ambient [CO_2_] of 380 µmol mol^−1^, relative humidity of 60–80%, temperature of 23 °C during the day and 16 °C during the night, and a photoperiod of 16 h. The response of net photosynthetic rate to CO_2_ and light were measured in four replicates. The light response curve was measured at an ambient CO_2_ level of 250 μmol mol^−1^ to promote photorespiratory conditions. We used these data for comparisons with the predicted responses of photosynthesis from the 3D C_4_ photosynthesis model developed here.

To reconstruct the 3D geometry (referred to as the ‘default’ geometry), plant tissues from the same set of maize plants measured for gas exchange and chlorophyll fluorescence as cited above were imaged using light microscopy (Olympus BX-51, at ×40 magnification). Sample preparation, fixation, and imaging were reported previously (Retta *et al.*, 2016b). A total of 120 leaf sections at 1-µm intervals were cut using one leaf from a sample that was least affected by the sample processing (Supplementary Fig. S1A, B). The images were first aligned using the 3D image-processing software Avizo Fire (VSG, France). The images included three veins each surrounded by a layer of bundle sheath cells, each of which was surrounded by a single layer of mesophyll cells forming a repetitive concentric arrangement of bundle sheath and mesophyll cells (Supplementary Fig. S1A). Therefore, using the symmetry of the geometry, only one concentric arrangement of mesophyll and bundle sheath cells along with the epidermis and IAS was considered by cropping the images to a representative region of interest, resulting in 3D images having a pixel resolution of 0.67 µm.

The regions of mesophyll and bundle sheath cells, chloroplasts, epidermis, IAS, and vascular bundle (Supplementary Fig. S1A) were labelled semi-automatically. Deformations of the epidermis on some of the images (Supplementary Fig. S1A) and missing sections were corrected for by interpolating through the consecutive images in Avizo. The chloroplasts of mesophyll cells were identified clearly thanks to their random arrangement, while numerous clustered chloroplasts of bundle sheath cells were segmented as a layer of centrifugally (towards mesophyll cells) arranged chloroplasts. Other microstructural features such as mitochondria, cytosol, and vacuole could not be identified due to insufficient resolution and contrast. Thus, mitochondria were assumed to be uniformly distributed in the cytosol, and vacuoles were created in MATLAB (version 7, The MathWorks, Inc., Natick, MA, USA) assuming the vacuole occupies 60% of the cell volume and accounting for sufficient cytosol volume required for CO_2_ hydration (Taiz, 1992). Two stomata identified on the light microscopy images were digitized as cylindrical pores. Two additional stomatal pores were created assuming a stomatal ratio of 1.0 so that the stomatal index matches that reported for maize (Driscoll *et al.*, 2006; Zheng *et al.*, 2013). The anatomical properties measured from the 3D geometry were compared with those reported specifically for maize or broadly for NADP-ME C_4_ subtype plants.

### Light propagation modeling and measurement of leaf optical properties

The propagation of light within the 3D geometry of maize leaf was modeled using a meshed Monte Carlo method (Watté *et al.*, 2015). Optical properties of mesophyll and bundle sheath cells were computed based on the Mie scattering theory from the distribution of organelles in these cells (Supplementary Tables S2, S3) (Aernouts *et al.*, 2014). The mean radius of organelles such as mitochondria, peroxisomes, nucleus, Golgi stack, and ribosome-like structures was calculated from the reported values of radius from the literature (Supplementary Tables S2). Using the mean radius and assuming spherical objects, a particle size distribution was calculated to estimate the number of organelles per unit volume. The latter was used to calculate the bulk scattering coefficients and anisotropy factor of cell media that were contributed by the optical properties of cell components such as mitochondria, peroxisomes, nucleus, Golgi stack, and ribosome-like structures (Supplementary Table S3). The scattering coefficient of chloroplasts was calculated from that of the grana. The thickness of the cell wall in our model was 160–190 nm, which is much less than the wavelength of light at which the Mie scattering theory is valid. Thus, the cell wall was not explicitly modeled but its scattering effect was accounted for by lumping the scattering coefficient of cell wall with that of the chloroplasts.

Absorption coefficents of the mesophyll and bundle sheath chloroplasts were calculated from the chlorophyll content of the leaf (Supplementary Table S3). The leaf chlorophyll content was assumed to be 539 µmol m^−2^, which is an average values of 501 µmol m^−2^ for maize having high nitrogen content and 579 µmol m^−2^, the maximum value reported for the NADP-ME subtype, for sorghum (Ghannoum *et al.*, 2005). The content of chlorophyll in bundle sheath cells can varry from 33% to 75% of the leaf chlorophyll content (Ghannoum *et al.*, 2005; Bellasio and Lundgren, 2016). It was assumed that the chlorophyll distribution was 50% in mesophyll and 50% in bundle sheath chloroplasts. The absorption coefficient of the epidermis, vacuole, and cytosol was set at 100 m^−1^ (Ho *et al.*, 2016).

The propagation of blue light (470 nm) and red light (665 nm) was modeled separately by assuming the illumination to be distributed uniformly over the top surface of the leaf. The resulting absorption profiles for red and blue light were combined with weighting fractions of 10% blue and 90% red (corresponding to the light source used during the gas exchange measurements) to derive an overall light absorption profile (Supplementary Table S3). The resulting light profile was then used to compute the potential rate of local electron transport and ATP and NADPH production in the mesophyll and bundle sheath chloroplasts (see ‘ATP and NADPH production model’ below).

To validate the light propagation model, light reflectance and transmittance were measured with the integrating spheres set-up elaborated by Aernouts *et al.* (2014), which was used previously by Ho *et al.* (2016). For light reflectance and transmission measurements, a different set of maize plants from another cultivar (cultivar P8057) were used as the measurement was not carried out at the same time as the gas exchange measurement. The plants were grown in a greenhouse at the facilities of Wageningen University & Research, The Netherlands. The set point for relative humidity during growth was 70% and that for temperature was 27 °C during the day and 22 °C during the night. The photoperiod was 12 h. These conditions were like the ones used to grow the maize plants for which the earlier-mentioned gas exchange data were acquired. In addition, cultivar P8057 received a total of N, P, K, and Mg of 1020, 710, 1630, and more than 160 mg per pot, respectively. Hybrid 2-02R10074A received 500 mg—1250 mg N per pot while total P, K, and Mg were 330, 1250, and 500 mg per pot, respectively (Retta *et al.*, 2016a). Therefore, the nitrogen applications, which influence chlorophyll content, were similar. The leaf thickness of a few randomly selected leaves was 200 ± 50 μm. Similarly, leaf thickness of hybrid 2-02R10074A ranged from 208 to 237 μm for the range of nitrogen treatments the cultivar received (Retta *et al.*, 2016b).

Reflectance and transmittance were measured from four plants using 2-mm samples from the mid-position of the maize leaves. The samples were placed between two integrating spheres and illuminated with a super continuum laser-based tunable light source to measure the total reflectance and transmittance in the wavelength range from 550 nm to 850 nm at 5 nm intervals. The measured total reflectance and transmittance were then compared with those computed with the light propagation model.

### Microscale gas exchange model

The microscale model for gas exchange of a C_4_ plant leaf was developed in analogy to the 3D R-D model for C_3_ leaves presented by Ho *et al.* (2016), and by extending a 2D R-D model of gas exchange of a C_4_ leaf (Retta *et al.*, 2016a). From the biochemical model of C_4_ photosynthesis (von Caemmerer and Furbank, 1999; Yin *et al.*, 2009; Yin and Struik, 2009), we developed the equations to account for 3D fluxes of CO_2_, bicarbonate, and O_2_ within the maize leaf and the rates of the associated processes. CO_2_ diffusion through the stomata was modeled by adjusting the effective diffusivity of CO_2_ through the stomatal pores to match the measured stomatal conductance without artificially changing stomatal geometry (Supplementary Fig. S2). Due to uncertainties of combining detailed metabolite pool concentrations with inhibition of enzyme activity by end-products, the inter-cell-type transport was assumed to be in a steady state and non-limiting in our model, as were the gas exchange measurements. Parameters of the 3D model are given in Table 1. Symbols, definitions, and units of the physical properties and photosynthetic parameters are given in Supplementary Table S1.

**Table 1.** Values of parameters used in the 3D model

Name	Symbol	Component	Value	Unit	References and notes
Diffusion coefficient of CO_2_	$D_{CO_{2}}$	Cell media	1.89 × 10^−9^/η	m^2^ s^−1^	Effective diffusivity (Frank *et al.*, 1996; Lide, 1999; Juurola *et al.*, 2005)
		IAS	1.57 × 10^−5^	m^2^ s^−1^	
		Cell wall	1.89 × 10^−9^ζ/η	m^2^ s^−1^	
Diffusion coefficient of O_2_	$D_{O_{2}}$	Cell media	1.97 × 10^−9^/η	m^2^ s^−1^	At 25 °C (Denny, 1993)
		IAS	2.10 × 10^−5^	m^2^ s^−1^	
		Cell wall	1.97 × 10^−9^ζ/η	m^2^ s^−1^	
Diffusion coefficient of HCO_3_^–^	$D_{HC{O_{3}}^{-}}$	Cell media	1.17 × 10^−9^/η	m^2^ s^−1^	Diffusion coefficient of bicarbonate in pure water at 25 °C (Geers and Gros, 2000)
Average thickness	*d*	Leaf tissue	165	µm	Measured from the 3D geometry
Fraction of e^−^ flux that follows the Q cycle		Chloroplast	1	Unitless	Yin and Struik (2012)
Number of H^+^ per mol ATP	*h*	Chloroplast	4	Unitless	Yin and Struik (2012)
Proton concentration		Cell media	10^–pH^	mol m^−3^	pH: 7.5 cytosol, 7.8 for chloroplast, 7.0 for vascular bundles, 4.0 for the vacuole (Evans *et al.*, 2009)
Henry’s constant for CO_2_		Cell media	0.83	Unitless	At 25 °C (Lide, 1999)
Henry’s constant for O_2_		Cell media	3.2×	Unitless	At 25 °C (Lide, 1999)
Light-saturated rate of e^−^ transport		Mesophyll chloroplast	138.43 ± 5.8	µmol m^−2^ s^−1^	Estimated
		Bundle sheath chloroplast	64.01 ± 5.8	µmol m^−2^ s^−1^	Estimated
Acid dissociation constant for H_2_CO_3_	*K*	Cell media	0.2	mol l^−1^	Jolly (1985), Ho *et al.* (2016)
Michaelis Menten constants of CA hydration		Mesophyll cytosol	2.8	mol m^−3^	Hatch and Burnell (1990)
Equilibrium constant for CA dehydration		Mesophyll cytosol	5.6×	mol m^−3^	Jolly (1985), Ho *et al.* (2016)
Michaelis–Menten constant of PEPC for bicarbonate		Mesophyll cytosol	2×	mol m^−3^	Bauwe (1986), Ho *et al.* (2016)
Michaelis–Menten constants of CA dehydration	$K_{HC{O_{3}}^{-}}$	Mesophyll cytosol	34	mol m^−3^	Pocker and Miksch (1978)
Michaelis–Menten constants of Rubisco for CO_2_		Bundle sheath chloroplast	485	µbar	Cousins *et al.* (2010)
Michaelis–Menten constants of Rubisco for O_2_		Bundle sheath chloroplast	146	mbar	Cousins *et al.* (2010)
CO_2_ hydration rate constant	*k*_1_	Cell media	3.9×	s^−1^	Jolly (1985), Ho *et al.* (2016)
CO_2_ dehydration rate constant	*k*_2_	Cell media	23	s^−1^	Jolly (1985), Ho *et al.* (2016)
Permeability to CO_2_ of membranes	$P_{CO_{2}}$	Bundle sheath	3.5 × 10^–3^	m s^−1^	Gutknecht *et al.* (1977)
		Chloroplast	1.75 × 10^–3^	m s^−1^	Half the membrane permeability
		Mesophyll	1.6 × 10^−2^	m s^−1^	Missner *et al.* (2008), assuming mesophyll plasma membrane is enriched with co-porins
Permeability to O_2_ of membranes	$P_{O_{2}}$	Chloroplast	1.25 × 10^−2^	m s^−1^	Ho *et al.* (2011), value halved for chloroplast envelope
		Mesophyll and bundle sheath	2.5 × 10^−2^	m s^−1^	
Rate of day respiration	$R_{d}$	Cell media	1.70 ± 0.27	µmol m^−2^ s^−1^	Retta *et al.* (2016b)
Rubisco specificity	$S_{C/O}$	Bundle sheath chloroplast	2862	Unitless	Cousins *et al.* (2010)
Lumped calibration factor	*s*ʹ	Bundle sheath chloroplast	0.325 ± 0.004	Unitless	Retta *et al.* (2016b)
Thickness of a component	*t*	Mesophyll cell wall Bundle sheath cell wall Plasmodesma	0.188 0.161 0.354	µm	Retta *et al.* (2016b), measured from electron microscopy images. Sum of cell wall thickness of mesophyll and bundle sheath
Maximum catalytic activity of CA	$V_{CA,max}$	Mesophyll cytosol	2.1 × 10^5^	µmol m^−3^ s^−1^	Pocker and Miksch (1978), Wang *et al.* (2014)
Maximum rate of Rubisco carboxylation	$V_{C,max}$	Bundle sheath chloroplast	78.5	µmol m^−2^ s^−1^	Retta *et al.* (2016b)
Maximum rate of PEP carboxylase	$V_{p,max}$	Mesophyll cytosol	183	µmol m^−2^ s^−1^	Retta *et al.* (2016b)
Fraction of ATP allocated to the C_4_ cycle	*x*	Mesophyll chloroplast	0.40	unitless	von Caemmerer and Furbank (1999)
Electron transport efficiency under limiting light for PSI	$\phi_{1,LL}$	Chloroplast	0.94	mol mol^−1^	Yin and Struik (2012), on the basis of light absorbed by PSI
Electron transport efficiency under limiting light for PSII	$\phi_{2,LL}$	Chloroplast	0.83	mol mol^−1^	Yin and Struik (2012), on the basis of light absorbed by PSI
Convexity index	θ	Chloroplast	0.97	Unitless	Assumed at the chloroplast level
Effective porosity	ζ	Mesophyll cell wall Bundle sheath cell wall Mesophyll–bundle sheath interface	0.5 0.1 3 × 10^–2^	Unitless	Evans (2021), the fraction of bundle sheath surface area occupied by plasmodesmata (Sowiński *et al.*, 2008)
Relative viscosity	η	Cell media	2	Unitless	Assumed

#### Biochemical model of C_4_ photosynthesis and hydration rates

The gross volumetric rate of CO_2_ fixation in bundle sheath chloroplasts, $V_{c}^{*}$, is given by the minimum of Rubisco-limited carboxylation rate (first term in brackets in Eq. 1), and electron-transport-limited carboxylation rate (second term in brackets in Eq. 1) based on ATP supply according to the biochemical model of C_4_ photosynthesis (von Caemmerer and Furbank, 1999; Yin and Struik, 2012):


$$\begin{array}{l} V_{c}^{*}=\min\left(\frac{[CO_{2}]\times V_{c,max}^{*}}{\left[CO_{2}]+K_{m,C}(1+[O_{2}]/K_{m,O}\right)},\frac{[CO_{2}]\times j_{ATP,C_{3}}^{*}}{3\;[CO_{2}]+7\gamma *[O_{2}]}\right) \end{array}$$ (1)

where *K*_m,C_ and *K*_m,O_ are Michaelis–Menten constants of Rubisco for CO_2_ and O_2_, respectively; γ^*^ = 0.5/*S*_C/O_, where *S*_C/O_ is the relative CO_2_/O_2_ specificity factor of Rubisco; $V_{c,max}^{*}$ is the volumetric rate of maximum capacity of Rubisco carboxylation (calculated using Eq. 13); and $j_{ATP,C_{3}}^{*}$ is the volumetric potential rate of ATP used for C_3_ cycle calculated by assuming 60% of the total ATP production ( Eq. 24) is allocated to the C_3_ cycle (von Caemmerer and Furbank, 1999). [CO_2_] and [O_2_] are the concentrations of CO_2_ and O_2_ in the bundle sheath chloroplasts, respectively.

The volumetric rate of PEP carboxylation ($V_{p}^{*}$) is given as a minimum of the enzyme-limited rate (first term in brackets in Eq. 2) and the electron-transport-limited rate of PEP carboxylation (second term in brackets in Eq. 2) (von Caemmerer and Furbank, 1999; Yin and Struik, 2012; Boyd *et al.*, 2015):


$$\begin{array}{l} V_{p}^{*}=\min\left(\frac{\;[\;HC{O_{3}}^{-}\;]\;\times V_{p,max}^{*}}{\;[\;HC{O_{3}}^{-}\;]\;+K_{h}},\frac{j_{ATP,C_{4}}^{*}}{2}\right) \end{array}$$ (2)

where *K*_h_ is the Michaelis–Menten constant of PEPC for bicarbonate and $V_{p,max}^{*}$ is the maximum catalytic rate of PEPC (calculated using Eq. 13).The volumetric rate of ATP used for the C_4_ cycle in mesophyll cells, $j_{ATP,C_{4}}^{*}$, was calculated by assuming 40% of the total ATP production (Eq. 24) is allocated to the C_4_ cycle (von Caemmerer and Furbank, 1999).

The net hydration of CO_2_ in the presence of CA, $B_{CA}$, is given by (Spalding and Portis, 1985; Tholen and Zhu, 2011):


$$\begin{array}{l} B_{CA}=\frac{V_{CA,max}^{*}\left([CO_{2}]-\frac{\left[HC{O_{3}}^{-}][H^{+}\right]}{K_{eq}}\right)}{K_{CO_{2}}+\frac{K_{CO_{2}}}{K_{HC{O_{3}}^{-}}}[HC{O_{3}}^{-}]+[CO_{2}]} \end{array}$$ (3)

where $V_{CA,max}^{*}$ is the maximum catalytic activity of CA per unit leaf volume; $\left[HC{O_{3}}^{-}\right]$ is the concentration of bicarbonate ions; $K_{CO_{2}}$ and $K_{HC{O_{3}}^{-}}$ are the Michaelis–Menten constants of CA hydration and dehydration, respectively; $K_{eq}$ is the equilibrium constant for CA; and [H^+^] is the concentration of $H^{+}$ ions.

Since bundle sheath cells of maize contain little CA, it is assumed that hydration of CO_2_ proceeds non-enzymatically in these cells (Burnell & Hatch, 1988). The net non-enzymatic hydration rate of CO_2_ per unit leaf volume, *B*_NCA_, is given by (Ho *et al.*, 2016):


$$\begin{array}{l} B_{NCA}=k_{1}[CO_{2}]-\frac{k_{2}[HC{O_{3}}^{-}][H^{+}]}{K} \end{array}$$ (4)

where *k*_1_ is the CO_2_ hydration rate constant; *k*_2_ is the CO_2_ dehydration rate constant; *K* is the acid dissociation constant for H_2_CO_3_; and [H^+^] is calculated from the pH of the cell compartments such as chloroplast stroma and cytosol. Equation 4 is assumed valid everywhere in the liquid phase.

#### CO_2_ transport inside a C_4_ leaf

The R-D equation for CO_2_ transport at steady-state is:


$$\begin{array}{l} \begin{array}{ll} & \nabla \times \;\left(D_{CO_{2}}\nabla [CO_{2}]\right)-G=0\\ & with\\ & G=B_{CA}+B_{NCA}-R^{*}\;in\;mesophyll\;cells\\ & \;=A_{n}^{*}-{\bar{V}}_{p}^{*}+B_{NCA}\;\;\;in\;bundle\;sheath\;cells\\ & \;\;\;=B_{NCA}-R^{*}\;\;in\;epidermis,\;vascular\;bundle\;\\ & \;\;\;=0\;\;\;\;\;\;in\;intercellular\;air\;spaces \end{array} \end{array}$$ (5)

where ∇ is the gradient operator; $D_{CO_{2}}$ is the diffusivity of CO_2_ in either liquid (cells) or IAS; *G* stands for source or sink terms in each cell component; $R^{*}$ is the volumetric rate of CO_2_ release through respiration in the cytosol of mesophyll and bundle sheath cells, epidermis and vascular bundles (Eqs 8, 9); $A_{n}^{*}$ is the volumetric rate of net photosynthesis which includes CO_2_ fixation in bundle sheath chloroplasts (Eq. 1) and CO_2_ release by respiration (Eq. 9) and photorespiration in bundle sheath cytosol (Eq. 10); and ${\bar{V}}_{p}^{*}$ is the average (indicated by the over score) rate of CO_2_ production through decarboxylation of C_4_ acids in the bundle sheath chloroplasts per unit leaf volume (Eq. 11). As in the standard C_4_ photosynthesis model (von Caemmerer and Furbank, 1999), we assume, the rate of decarboxylation is equal to the rate of PEP carboxylation, so the same symbol, ${\bar{V}}_{p}^{*}$, is used.

The diffusion of bicarbonate ions in epidermis, mesophyll cells, vasculature, or bundle sheath cells is collectively given by (Retta *et al.*, 2016a):


$$\begin{array}{l} \nabla \times \;\left(D_{HC{O_{3}}^{-}}\nabla \left[HC{O_{3}}^{-}\right]\right)+B_{CA}+B_{NCA}-V_{p}^{*}=0 \end{array}$$ (6)

where $D_{HC{O_{3}}^{-}}$ is the diffusivity of bicarbonate in the respective cell compartments. *B*_CA_ and $V_{p}^{*}$ is applied only to the mesophyll cytosol (Eqs 2, 3).

#### O_2_ transport inside a C_4_ leaf

The steady state R-D equation for O_2_ transport is given by:


$$\begin{array}{l} \nabla \times \;\left(D_{O_{2}}\nabla \left[O_{2}\right]\right)+E_{O_{2}}^{*}-r^{*}-R^{*}=0 \end{array}$$ (7)

where $D_{O_{2}}$ is the diffusivity of O_2_; $E_{O_{2}}^{*}$ is the volumetric rate of oxygen production by chloroplasts of mesophyll or bundle sheath cells and zero elsewhere; and $r^{*}$ is the total O_2_ consumption due to RuBP oxygenation during photorespiration in the bundle sheath chloroplast, and additional consumption of O_2_ (0.5 mol of O_2_ per RuBP oxygenation) by glycolate oxidase in peroxisomes in the photorespiratory cycle (Eq. 12) (Somerville, 2001). The rate of oxygen evolution, $E_{O_{2}}^{*}$, associated with the linear electron transport in chloroplasts was set to be equal to a quarter of the rate of linear electron flux (Eq. 19), because the production of 1 mol of O_2_ is accompanied by the flow of four electrons.

#### Calculation of microscale model variables

Respiratory CO_2_ release is assumed to occur in the epidermis, cytosol of mesophyll and bundle sheath cells, and the phloem tissue in the vascular bundles. Rate of respiratory CO_2_ release in each of these components is calculated by assuming that the epidermis is as metabolically active as mesophyll, bundle sheath, or vascular bundles. Volumetric rate of respiratory CO_2_ release in the epidermis (EPI), ${R_{EPI}}^{*}$, is given by:


$$\begin{array}{l} {R_{EPI}}^{*}=\frac{R_{d}}{d\times f_{resp}} \end{array}$$ (8)

where *R*_d_ is the rate of day respiration; *f*_resp_ is the sum of volume fractions of vascular bundles, EPI, and cytosol of mesophyll and bundle sheath cells; and *d* is the average thickness of leaf tissue.

Volumetric rate of respiratory CO_2_ release in component *i* (mesophyll, bundle sheath, or vascular bundles), ${R_{i}}^{*}$, is given by:


$$\begin{array}{l} {R_{i}}^{*}=\frac{R_{d}}{d\times f_{resp}\times f_{i}} \end{array}$$ (9)

where *f*_*i*_ is the volume fraction of a component *i* in the leaf tissue where the rate is applicable.

The volumetric rate of photorespiratory CO_2_ release in bundle sheath cytosol, $r_{p,CO_{2}}^{*}$, is given by:


$$\begin{array}{l} r_{p,CO_{2}}^{*}=\frac{\int_{V_{BS,ch}}V_{c}^{*}\frac{\gamma *[O_{2}]}{\left[CO_{2}\right]}dv}{\int_{V_{BS,cy}}(\cdot )dv} \end{array}$$ (10)

where $V_{c}^{*}$ is given by Eq. 1; *V*_BS,ch_ is the volume of bundle sheath chloroplast; and *V*_BS,cy_ is the volume of the cytosol of bundle sheath. Volume of bundle sheath cytosol is calculated by $\int_{V_{BS,cy}}(\cdot )dv=∭dxdydz$

The average rate of CO_2_ production from decarboxylation of C_4_ acids, assumed to proceed at the same rate as PEP carboxylation (Eq. 2) in mesophyll cytosol (subscript M,cy) (von Caemmerer and Furbank, 1999), was calculated as:


$$\begin{array}{l} {\bar{V}}_{p}^{*}=\frac{\int_{V_{M,cy}}V_{p}^{*}dv}{\int_{V_{BS,ch}}(\cdot )dv} \end{array}$$ (11)

where *V*_BS,ch_ is the volume of bundle sheath chloroplast (subscript BS,ch) and *V*_M,cy_ is the volume of mesophyll cytosol.

Consumption of 1 mol of O_2_ by photorespiration results in the release of 0.5 mol of CO_2_. In addition, 0.5 mol O_2_ is consumed per RuBP oxygenation by glycolate oxidase in peroxisomes in the photorespiratory cycle. Thus, the volumetric rate of oxygen consumption by photorespiration, $r_{p,O_{2}}^{*}$, in a bundle sheath chloroplast is given by:


$$\begin{array}{l} r_{p,O_{2}}^{*}=\frac{\int_{V_{BS,ch}}3V_{c}^{*}\frac{\gamma *[O_{2}]}{\left[CO_{2}\right]}dv}{\int_{V_{BS,ch}}(\cdot )dv} \end{array}$$ (12)

where the volume of bundle sheath chloroplast is calculated by $\int_{V_{BS,ch}}(\cdot )dv=∭dxdydz$.

Volumetric rates (marked with asterisk) of leaf area-based quantity *Q* such as *V*_c,max_ or *V*_p,max_ was calculated as:


$$\begin{array}{l} Q^{*}=\frac{Q}{d\times f_{i}} \end{array}$$ (13)

where *f*_*i*_ is the volume fraction of a component *i* (mesophyll cytosol or bundle sheath chloroplast) in the leaf tissue where the rate is applicable.

The rate of net photosynthesis was calculated by integrating the flux of CO_2_ over the outer epidermis surfaces. The computed photosynthesis, $A_{n}$, is given as:


$$\begin{array}{l} A_{n}=\frac{\int_{S_{EPI}}\left|Flux\right|dA}{S_{leaf}} \end{array}$$ (14)

where Flux is a normal diffusive flux defined as n(−DΔC); **n** is the normal vector of unit length; *S*_EPI_ is the epidermis surface; and *S*_leaf_ is a projected area of the leaf.

The rate of CO_2_ leakage, *L* is given by:


$$\begin{array}{l} L={\bar{V}}_{p}-A_{n}-R_{M} \end{array}$$ (15)

where ${\bar{V}}_{p}$ is the mean rate of PEP carboxylation calculated from Eq. 2 and $R_{M}$ is the rate of CO_2_ release in mesophyll cytosol through respiration (Eq. 9).

The leakiness Φ is calculated as:


$$\begin{array}{l} \Phi \;=\;\frac{L}{{\bar{V}}_{p}} \end{array}$$ (16)

#### Calculation of fluxes and resistances to transport in boundaries

The flux of CO_2_, $HC{O_{3}}^{-}$, and O_2_ at the boundary of cuticle, mesophyll, or bundle sheath cells is given by:


$$\begin{array}{l} J_{y}=-P_{y}\Delta \left[y\right] \end{array}$$ (17)

where *y* stands for CO_2_, O_2_, and $HC{O_{3}}^{-}$; *P*_*y*_ is the permeability of cuticle, plasma membrane, chloroplast envelope, cell wall (CO_2_ and O_2_) or the mesophyll–bundle sheath interface to *y*; and Δ[y] is the difference in concentration of *y* across each boundary.

*P*_*y*_ is given by:


$$\begin{array}{l} P_{y}=\frac{D_{y}\zeta }{t} \end{array}$$ (18)

where *D*_*y*_ is the diffusion coefficient of *y* in a cell component; ζ is the effective porosity; and *t* is the thickness of diffusion path. For the plasmodesmata, *D*_*y*_ is assumed equal to that in the cytosol; ζ is the ratio of the bundle sheath surface area covered by plasmodesmata to the total surface area of bundle sheath; and *t* is the length of the plasmodesmata. At the outer tangential cell wall of bundle sheath cells of maize, a suberin lamella is deposited (Hattersley and Browning, 1981). We assume that gas exchange occurs only through plasmodesmata at the mesophyll–bundle sheath interface. Thus, an insulated boundary condition was assumed at the surface of the bundle sheath cells exposed to the IAS.

### ATP and NADPH production model

The ATP production was modeled to be driven by local light intensity at the level of the chloroplast using the light profile of the 3D geometry. The model of ATP production is explained in detail by Yin and Struik (2018). The model for electron transport and ATP production aimed to ensure that the ATP and NADPH requirements of C_4_ and C_3_ cycles were met at the whole-leaf level. A brief description of the model with selected equations relevant for this study is given below.

The volumetric rate of linear electron transport (LET) in mesophyll or bundle sheath chloroplasts, $j_{i}^{*}$ is given by a commonly used non-rectangular hyperbolic model (Farquhar and Wong, 1984; Yin *et al*., 2006):


$$\begin{array}{l} j_{i}^{*}=\frac{\alpha_{2,LL,i}I_{abs,i}^{*}+j_{\max,i}^{*}-\sqrt{{\left(\alpha_{2,LL,i}\;I_{abs,i}^{*}+j_{\max,i}^{*}\right)}^{2}-4\theta \;\alpha_{2,LL,i}\;I_{abs,i}^{*}\;j_{\max,i}^{*}}}{2\;\theta } \end{array}$$ (19)

where *i* refers to either mesophyll or bundle sheath chloroplasts; $\alpha_{2,LL,i}$ is the photochemical efficiency of linear electron transport of photosystem II (PSII) under limiting light (LL) (based on light absorbed by both photosystems) when cyclic electron transport (CET) occurs simultaneously; $I_{abs,i}^{*}$ is the rate of photon absorption by mesophyll or bundle sheath chloroplasts per unit leaf volume, calculated by the product of actinic irradiance (*I*_inc_) and the fraction of that irradiance absorbed by the chloroplasts in these cells expressed per volume; $j_{\max,i}^{*}$ is the maximum volumetric electron transport rate under saturating illumination (calculated using Eq. 13); and θ is the convexity index.

$\alpha_{2,LL,i}$ is calculated as (Yin *et al*., 2006):


$$\begin{array}{l} \alpha_{2,LL,i}=\frac{\phi_{2,LL}\;\left(1-f_{CET,i}\right)}{\phi_{2,LL}/\phi_{1,LL}+\;\left(1-f_{CET,i}\right)} \end{array}$$ (20)

where $\phi_{1,LL}$ and $\phi_{2,LL}$ are the electron transport efficiencies under limiting light for photosystem I (PSI) and PSII, respectively (unitless, on the basis of light absorbed by each photosystem). $f_{CET,i}$ is the fraction of CET in mesophyll or bundle sheath chloroplasts given by:


$$\begin{array}{l} f_{CET,i}=\frac{\int_{i}j_{LL,CET,i}^{*}\;dv}{\int_{i}j_{LL,CET,i}^{*}\;dv\;+\;\int_{i}j_{LL,LET,i}^{*}\;dv} \end{array}$$ (21)

where $j_{LL,CET,\;i}^{*}$ is the light-limited rate of CET, and $j_{LL,LET,\;i}^{*}$ is the light-limited rate of LET in either mesophyll or bundle sheath.

$j_{LL,CET,\;i}^{*}$ and $j_{LL,LET,\;i}^{*}$ are calculated as (Yin and Struik, 2012):


$$\begin{array}{l} j_{LL,LET,i}^{*}\;=\;\frac{\phi_{2,LL}}{1+\phi_{2,LL}/\phi_{1,LL}\;}\;u_{i}\;I_{abs,i}^{*} \end{array}$$ (22)

$$\begin{array}{l} j_{LL,CET,i}^{*}\;=\;\phi_{1,LL}\;\left(1-u_{i}\right)\;I_{abs,i}^{*} \end{array}$$ (23)

where *u*_*i*_ is the fraction of light for LET in mesophyll or bundle sheath chloroplasts, which were calculated according to (Yin and Struik, 2018).

The total ATP production is the sum of ATP production in mesophyll and in bundle sheath chloroplasts. The ATP production rate per leaf area basis in mesophyll or bundle sheath chloroplasts, $j_{ATP,i}$, can be formulated as a function of PSII LET flux as (Yin and Struik, 2012):


$$\begin{array}{l} j_{ATP,i}=\left(\frac{2\;+f_{Q}-f_{CET,i}}{h\;\left(1-f_{CET,i}\right)}\right)\frac{1}{S_{leaf}}\int_{i}j_{i}^{*}dv \end{array}$$ (24)

where *h* is the number of H^+^ ions required to synthesize 1 ATP; $f_{Q}$ is the fraction of the electron flux that follows the Q cycle (Yin and Struik, 2012); and $S_{leaf}$ is the area of a cross section of the computational domain. So, the term $\frac{1}{S_{leaf}}\int_{i}j_{i}^{*}dv$ represents the volumetrically integrated rate of LET per leaf area basis.

The rate of whole-leaf total ATP production calculated from chlorophyll fluorescence (CF) data, *j*_ATP,CF_, was given by (Yin *et al.*, 2011):


$$\begin{array}{l} \begin{array}{ll} & j_{ATP,CF}=\frac{s^{\prime }I_{inc}\left(\Delta F/F{{}^{\prime }}_{\;\;\;m}\right)}{1-x}\\ & \Delta F/F{{}^{\prime }}_{\;\;\;m}=\left(F{{}^{\prime }}_{\;\;\;m}-F_{s}^{}\right)/F{{}^{\prime }}_{\;\;\;m} \end{array} \end{array}$$ (25)

where *s*ʹ is the lumped calibration factor; *F*_s_ is the steady-state relative fluorescence yield; *F*ʹ_m_ is the maximum relative fluorescence yield in the leaf; and *x* is the fraction of ATP allocated to the C_4_ cycle. For this calculation, a constant *x* of 0.40, which is shown to be an optimal distribution for high photosynthesis, for all irradiances was assumed (von Caemmerer and Furbank, 1999; Yin *et al.*, 2011; Yin and Struik, 2012). This value changes at low light intensity (Kromdijk *et al.*, 2010). However, accounting for such change requires a detailed description of the kinetics of photosynthesis and exchange of metabolites, which is beyond the scope of our study (Bellasio, 2017).

The potential ATP production rate ($j_{ATP}$) computed for the whole leaf level is given by:


$$\begin{array}{l} j_{ATP}=\frac{\int_{V_{leaf}}j_{ATP}^{*}dv}{S_{leaf}} \end{array}$$ (26)

where *S*_leaf_ is the area of a cross section of the computational domain; j^*^_ATP_ is the local volumetric rate of ATP production determined from Eq. 24; and *V*_leaf_ is the total leaf volume.

The light saturated electron transport rates in mesophyll and bundle sheath cells, $j_{\max,M}$ and $j_{\max,BS}$ (Eq. 19), were fitted by minimizing the difference between the respective ATP production rate determined from chlorophyll fluorescence measurement (Eq. 25) and that modeled from the potential electron transport rate at the whole leaf level (Eq. 26). To avoid overfitting, it was assumed that:


$$\begin{array}{l} \frac{j_{\max,BS}}{j_{\max,M}}=\frac{\alpha_{2LL,BS}I_{abs,BS}}{\alpha_{2LL,M}I_{abs,M}} \end{array}$$ (27)

where $\alpha_{2LL,BS}$ or $\alpha_{2LL,M}$ is given by Eq. 20; $I_{abs,BS}$ is the absorbed irradiance by bundle sheath chloroplasts; and $I_{abs,M}$ is the absorbed irradiance by mesophyll chloroplasts.

The rate of NADPH production per leaf area in mesophyll or bundle sheath chloroplasts, $j_{NADPH,i}$, is given by:


$$\begin{array}{l} j_{NADPH,i}=\frac{0.5}{S_{leaf}}\int_{i}j_{i}^{*}dv \end{array}$$ (28)

where 0.5 refers to 0.5 mol NADPH produced per mol electrons transferred by LET.

The fraction ATP in bundle sheath cells ($f_{ATP,BS}$) is calculated as:


$$\begin{array}{l} f_{\mathrm{ATP},BS}=\frac{j_{ATP,BS}}{j_{ATP,M}+j_{ATP,BS}} \end{array}$$ (29)

where $j_{ATP,BS}$ and $j_{ATP,M}$ are given by Eq. 24.

The fraction of NADPH in bundle sheath cells ($f_{NADPH,BS}$) is calculated as:


$$\begin{array}{l} f_{NADHP,BS}=\frac{j_{NADPH,BS}}{j_{NADPH,BS}+j_{NADPH,M}} \end{array}$$ (30)

where $j_{NADPH,BS}$ and $j_{NADPH,M}$ are given by Eq. 28.

### Modeling chloroplast arrangement

The chloroplast arrangements were modeled based on microscopic images of chloroplast movement for maize reported previously (Yamada *et al.*, 2009; Maai *et al.*, 2020b) assuming the vacuole allows free movement of chloroplasts. From the default geometry (Supplementary Fig. S1C), two leaf geometries were developed to simulate the aggregative movement and the avoidance movement of mesophyll chloroplasts (Supplementary Fig. S2D, E). The key morphological operations in MATLAB software (R2017b) used to model chloroplast arrangements were ‘dilation’, which adds pixels to the boundary of a domain, and ‘erosion’, which removes pixels from the boundary of a domain. The domain of bundle sheath cells of the default anatomy (Supplementary Fig. S2C) was dilated to intersect the domain of mesophyll cells in contact with the bundle sheath cells to model the aggregative movement of mesophyll chloroplasts (Supplementary Fig. S2D). The domain created as the result of the intersection was considered as the chloroplast domain (Supplementary Fig. S2D) after it was eroded in MATLAB and the volume fraction of chloroplasts per cell matched reasonably well with that of the default geometry (Supplementary Fig. S3). To model the avoidance movement of mesophyll chloroplasts (Supplementary Fig. S2E), a chloroplast layer was similarly made but only for those mesophyll cells lying between veins.

### 3D leaf anatomy, chloroplast arrangement, and ATP and NADPH production

The 3D model of light propagation was coupled to the model of ATP and NADPH production (Eqs 19–24, 28) to determine the total ATP and NADPH production. The total ATP was partitioned between C_3_ and C_4_ cells at cell level. Forty percent of the total produced ATP (Eq. 24) was assumed to be used for the C_4_ cycle (von Caemmerer and Furbank, 1999). Then, the gas transport models (Eqs 5–7) were solved using the 3D structure for various chloroplast arrangement scenarios. The responses of *A*_n_ (Eq. 14) to *I*_inc_ and to intercellular CO_2_ (*C*_i_) were computed and compared with those obtained from the gas exchange measurement. The fractions of CET in bundle sheath cells (Eq. 21) and the fraction of ATP and NADPH production in bundle sheath chloroplasts (Eqs 29–30) calculated from the default geometry were compared with cases of chloroplast arrangement.

### Comparison of a 2D and a 3D model of gas transport

Three 2D slices from the 3D geometry were taken from the positions 30, 60, and 90 of 120 slices to directly compare the differences in modeled photosynthesis on the same leaf using the same R-D approach between the simpler 2D geometry and the corresponding 3D geometry (Supplementary Fig. S4). The light absorption profile and the mean ATP production rate were set equivalent to those of the 3D geometry at the positions where the slices were made. Following Retta *et al.* (2016a), we assumed that the concentration of CO_2_ in the IAS was uniform for the 2D model. Therefore, the gas concentrations at the exposed surface of mesophyll were set equal to the mean concentrations in IAS of the 3D model. Retta *et al.* (2016a) also assumed that the diffusion coefficients of CO_2_ and bicarbonate were equal to those of pure water in order to improve the match between the measured and simulated gas exchange data. Thus, we also solved the 2D model from slice 60 with these assumptions so that the relative viscosity of the cell cytosol (η) was assumed to be 1.0 or 2.0, where the latter value is assumed closest to the biological medium. All other parameter values were kept the same as the ones reported in Table 1. The response of *A*_n_ to *C*_i_ was computed for the 2D model (for various assumption of η) and compared with the results obtained from the 3D model.

### Numerical solution

A 3D leaf tissue of 124 × 124 × 200 µm was represented by 9.428×10^6^ cube elements with a length of 0.67 µm. The lateral sides of this geometry were assumed to have no net flux, while external concentrations of CO_2_ and O_2_ were assumed at the adaxial and abaxial sides. The R-D equations were discretized over the finite volume grid and the resulting linear systems of algebraic equations of the unknown concentrations at the volume nodes were solved by the preconditioned conjugate gradient procedure in MATLAB using the finite volume method (Versteeg and Malalasekera, 1995; Ho *et al.*, 2016). We used a 16-GB RAM node of the high-performance computer in the VSC-Flemish Supercomputer Center (Belgium) to run the model. The computation time was 72 h.

## Results

### The 3D leaf structure impacts gas transport and light absorbance

**Fig. 1.**
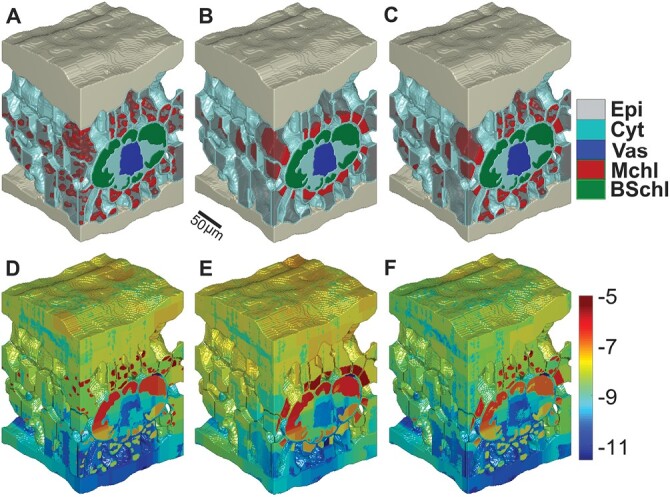
Three-dimensional leaf anatomy of a maize leaf and the various chloroplast arrangements. The geometries were obtained for the epidermis (Epi), chloroplasts of mesophyll (Mchl) and bundle sheath (BSchl) cells, vacuole (Vac), vascular bundle (Vas), and cytosol (Cyt). (A–C) Default geometry (A), aggregative movement of mesophyll chloroplasts (B), and geometry for avoidance movement (C). (D–F) Light absorbance for the default geometry (D), aggregative movement of mesophyll chloroplasts (E), and avoidance movement of mesophyll chloroplasts (F). The log10 of absorbance is shown in the color bar. Leaf tissue dimensions are 124 × 124 × 200 µm. Scale bar: 50 µm.

The 3D geometry (Fig. 1A) consisted of the epidermis, stomata, IAS, mesophyll cells, bundle sheath cells and vasculature, as main components of the maize leaf anatomy. Leaf anatomical characteristics measured from this geometry were compared with literature values (Table 2). The anatomical features such as exposed surface area of mesophyll per leaf area, surface area of bundle sheath cells per leaf area, surface to volume ratio of mesophyll and bundle sheath cells and volume fraction of chloroplasts in mesophyll and bundle sheath compared well with literature. Table 2 shows that the volume per leaf area of mesophyll and bundle sheath cells of the 3D geometry were lower than those reported in the literature.

**Table 2.** Anatomical properties of the 3D geometrical model in comparison with literature values

Anatomical trait	3D model	Literature values	References and notes
Mesophyll			
Cell volume fraction (m^3^ m^−2^)	30.2% 52.2	47% 80–120	Measured from 2D images, NADP-ME (Dengler *et al.*, 1994) Volume per leaf area, NADP-ME (Pignon *et al.*, 2019)
Chloroplast volume fraction (m^3^ m^−2^)	13 × 10^−6^	10 × 10^−6^–14 × 10^−6^	Volume per leaf area, NADP-ME (Pignon *et al.*, 2019)
Surface to volume ratio (m^2^ m^−3^)	0.14 × 10^6^	0.12 × 10^6^	Measured from 2D images, NADP-ME (Dengler *et al.*, 1994)
Exposed surface per leaf area, *S*_M_ (m^2^ m^−2^)	12	6–15	Values for C_4_ plants including NADP-ME (Dengler *et al.*, 1994; von Caemmerer *et al*., 2007; El-Sharkawy, 2009; Retta *et al.*, 2016b; Sonawane *et al.*, 2021)
Bundle-sheath			
Cell volume fraction (m^3^ m^−2^)	16.7% 27.6	12% 35–45	NADP-ME (Dengler *et al.*, 1994) Volume per leaf area, NADP-ME (Pignon *et al.*, 2019)
Chloroplast volume fraction (m^3^ m^−2^)	15 × 10^−6^	6 × 10^−6^–10 × 10^−6^	Volume per leaf area, NADP-ME (Pignon *et al.*, 2019)
Surface to volume ratio (m^2^ m^−3^)	0.045 × 10^6^	0.01 × 10^−6^–0.050 × 10^6^	2D, NADP-ME (Dengler *et al.*, 1994)
Surface area per unit leaf area, *S*_BS_ (m^2^ m^−2^)	1.9	0.6–3.1	NADP-ME (von Caemmerer *et al*., 2007; Retta *et al.*, 2016b; Sonawane *et al.*, 2021)
Intercellular airspace			
Volume fraction (%)	22.3	20–40	NADP-ME (Dengler *et al.*, 1994; Retta *et al.*, 2016b; Sonawane *et al.*, 2021)

**Fig. 2.**
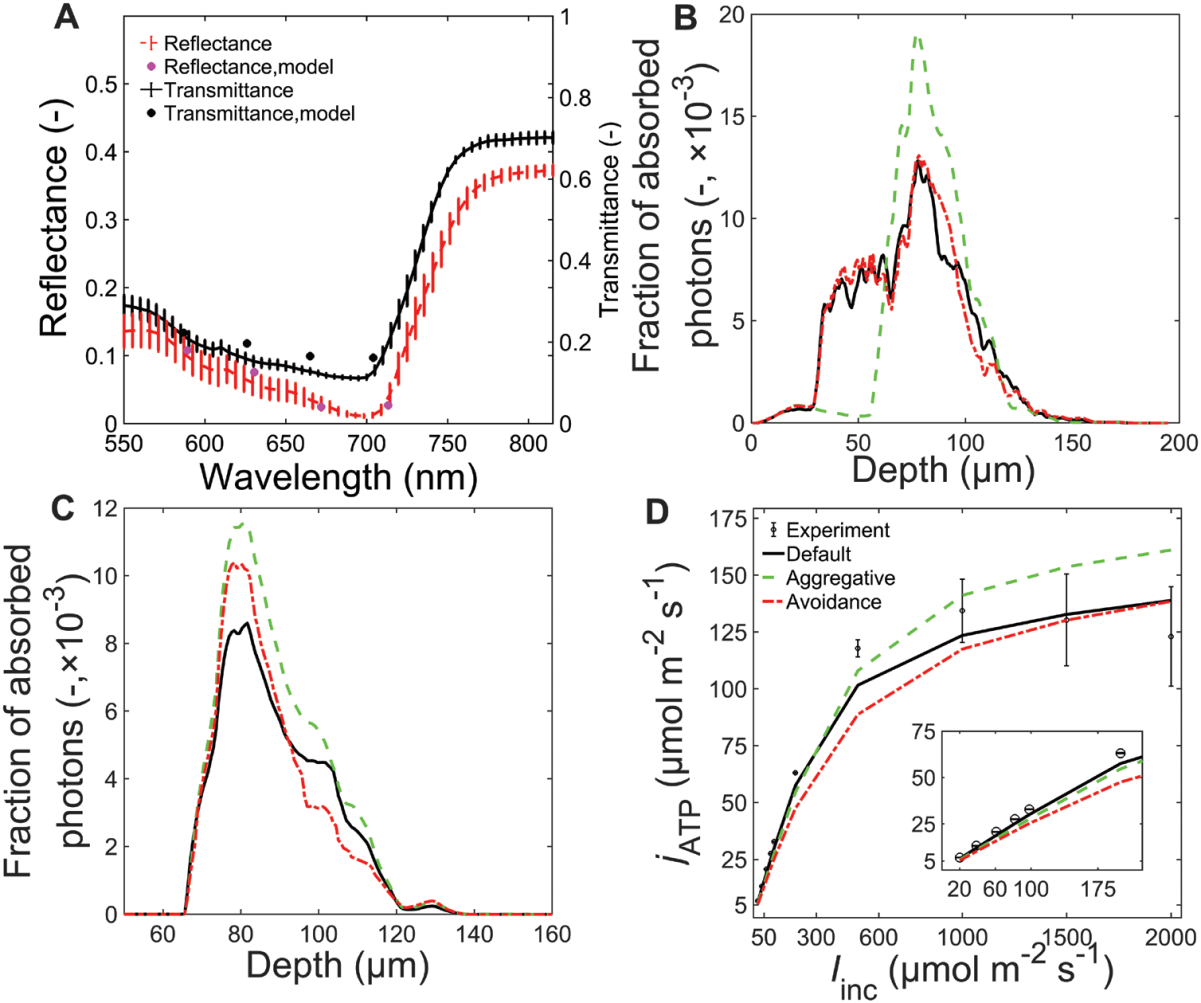
Validation of light propagation model and the roles of chloroplast arrangement on light propagation and ATP production. (A) Comparison of measured (symbol) and simulated (lines) reflectance (dashed line and pink circle) and transmittance (solid line and black circles) spectra for four maize leaves (*Zea mays* L.) in the default mesophyll chloroplasts case. (B–D) Simulated fraction of absorbed photons across the leaf depth (B) and just across the depth zone of bundle sheath cells (C) and the response of ATP production to irradiance (*I*_inc_) (D) for the cases of default geometry (solid line), aggregative movement of mesophyll chloroplasts (dashed green line) and the avoidance movement of mesophyll chloroplasts (dashed-dotted red line). The symbols in (D) show the ATP production calculated from experimental data. Inset in (D) shows the total ATP production at low light intensities.

**Fig. 3.**
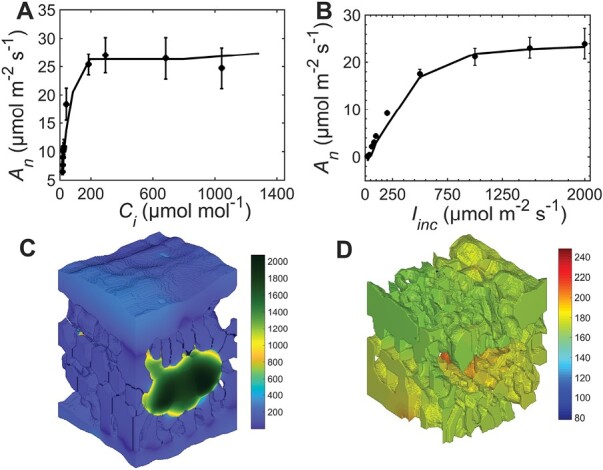
Comparison of model prediction of the response of net photosynthesis (*A*_n_) and CO_2_ profile in leaf tissue. (A, B) The response of *A*_n_ to intercellular CO_2_ (*C*_i_) (A) and to irradiance (*I*_inc_) (B). Model predictions are shown by solid lines and experimental data are shown by symbols with error bars. Simulation conditions were irradiance of 1500 µmol m^−2^ s^−1^, ambient CO_2_ of 380 µmol mol^−1^, and oxygen of 210 mmol mol^−1^ for (A). The response to *I*_inc_ (B) was computed at ambient [CO_2_] of 250 µmol mol^−1^ and 210 mmol mol^−1^ O_2_. (C, D) The profiles of CO_2_ concentration (µmol mol^−1^) in the liquid phase (C) and in the intercellular airspace (D). In (A) and (B), the bars show standard error (*n* = 4). Color bars (C, D) are the CO_2_ concentrations (µmol mol^−1^).

Figure 2A shows the comparison of optical properties measured experimentally with those predicted from the light propagation model using the default mesophyll chloroplasts configuration. The root mean square errors for the prediction of spectra of optical properties were 4% and 10% for total reflectance and transmittance (Fig. 2A), respectively. The simulation model for light propagation thus gave reasonable predictions for the reflectance and transmittance of red light (which was 90% of the total applied light in the gas exchange measurement, Fig. 2A). The fraction of photon energy absorbed by the leaf (Fig. 2B, C), as illustrated by a 3D representation in Fig. 1D, shows that halfway through the leaf depth, the photon absorption was enhanced by the chloroplasts within the bundle sheath cells. There was little effect of changing the scattering coefficient of chloroplasts by 25% on the absorption profile suggesting the effectiveness of our approach of accounting the scattering property of cell wall with that of chloroplasts. The response of *j*_ATP_ to *I*_inc_ (Fig. 2D) was computed using the estimated maximum rate of electron transport (Table 1). The light model predicts that 87% was absorbed by the leaf, while the mesophyll chloroplasts absorbed 55% and the bundle sheath chloroplasts absorbed 28% (Table 3). The predicted responses of *A*_n_ to changes in *I*_inc_ and *C*_i_ are shown in Fig. 3A, B. The mean square percentage error for the *A*_n_*–I*_inc_ curve was 6.5% and that of the *A*_n_*–C*_i_ curve was 1.8%. The deviations at low light intensity contributed to the higher mean square percentage error of the *A*_n_*–I*_inc_ curve. At high light intensity where the chloroplast movement scenarios were considered, the error in the prediction was much lower (Fig. 3). Thus, the model could reasonably predict the response curves. The mean [CO_2_] in the bundle sheath chloroplast (*C*_c_) was about five times higher than ambient [CO_2_] (380 µmol mol^−1^) when computed at 210 mmol mol^−1^ O_2_ and *I*_inc_ of 1500 µmol m^−2^ s^−1^ due to the CCM (Fig. 3C). The predicted CO_2_ concentration profile in the IAS was largely homogeneous (Fig. 3D).

**Table 3.** Effect of chloroplast arrangement on light absorbance, ATP production, and NADPH production

Parameter	Default	Aggregative movement of mesophyll chloroplasts	Avoidance movement of mesophyll chloroplasts
Total absorbance	0.87	0.81	0.82
Chloroplast absorbance	0.84	0.77	0.71
Transmittance	0.051	0.10	0.10
Absorbance, bundle sheath:mesophyll	0.51	0.88	0.74
$u_{M}$ ^*a*^	0.89	0.99	0.96
$u_{BS}$ ^*b*^	0.28	0.33	0.31
$f_{CET,BS}$ ^*c*^	0.85	0.81	0.83
$f_{CET,M}$ ^*d*^	0.20	0.01	0.08
$f_{NADPH,BS}$ ^*e*^	0.14	0.23	0.20
$f_{ATP,BS}$ ^*f*^	0.39	0.53	0.49

^*a*^ Fraction of light for LET in mesophyll cells.

^*b*^ Fraction of light for LET in bundle sheath cells.

^*c*^ Fraction of CET in bundle sheath.

^*d*^ Fraction of CET in mesophyll.

^*e*^ Fraction of NADPH produced in bundle sheath cells.

^*f*^ Fraction of ATP produced in bundle sheath.

The 3D model was compared with a simpler 2D model (Retta *et al.*, 2016a) using random slices from the same default leaf geometry (Supplementary Fig. S4). The *A*_n_*–C*_i_ curve was underestimated by the 2D models, especially if the viscosity of the aqueous symplast was estimated as double that of pure water (Supplementary Fig. S4A). The *C*_c_ estimated from the 3D model was 1584 µmol mol^−1^ (ambient [CO_2_] of 380 µmol mol^−1^, 210 mmol mol^−1^ O_2_, and *I*_inc_ of 1500 µmol m^−2^ s^−1^). The *C*_c_ calculated using three 2D slices selected at *z*-positions of 20, 40, and 60 in the stack of 120 slices was 1520 ± 270 µmol mol^−1^ suggesting that if *C*_c_ estimated from the 3D model is the more accurate, the use of randomly selected 2D slices will bias the concentration of CO_2_ at the sites of Rubisco. In particular, the 3D model of light absorption within the leaf structure profile (Fig. 1D) represents a major improvement over the assumption of constant photon flux across the leaf in the model of Retta *et al.* (2016a). Moreover, 2D models understate the interconnectivity of the IAS prevalent in the 3D space and may lead to areas of IAS not connected to a stoma, which compromises the modeling of CO_2_ diffusion within the IAS (Supplementary Fig. S4). Therefore, the 3D model is definitely more accurate than the 2D model to simulate diffusion of gases and photosynthesis in a C_4_ leaf.

### Chloroplast movement increased the relative light absorbance by bundle sheath chloroplasts

wThe effect of mesophyll and bundle sheath chloroplast arrangement on light propagation is illustrated in Fig. 1D–F. The geometries of the different chloroplast arrangement scenarios (Fig. 1B, C) have similar volume fractions of chloroplasts per cell and total volume fractions of chloroplasts as the default geometry (Fig. 1A; Supplementary Fig. S3). The interveinal mesophyll cells whose chloroplast arrangement changed accounted for 25% of the mesophyll volume and the total chloroplast in avoidance arrangement is 18% of the total chloroplast volume.

Table 3 shows the total light absorption by the leaf decreased by ca. 7% for aggregative movement of mesophyll chloroplasts and ca. 6% for avoidance movement compared with that of the default geometry. The avoidance movements reduced the absorbance of chloroplasts by ca. 15% while aggregative movement resulted in ca. 8% lower absorbance. On the other hand, the transmittance of light through the maize leaf doubled in both chloroplast movement scenarios (Table 3). By contrast, the ratio of absorbed light by bundle sheath chloroplasts relative to mesophyll chloroplasts was increased by ca. 73% over the default case due to the aggregative movement and ca. 45% due to avoidance movement. Although transmittance did not change (Table 3) the reflectance increased by 6% in the avoidance movement compared with the aggregative movement.

The total leaf absorbance profile across the leaf depth is shown in Fig. 2B. The profile for the avoidance movement slightly differed close to mid-leaf thickness from that of the default case. The profile for avoidance movement changed in such a way that there was an increased absorbance followed by steeper decline starting at 100 µm depth, which was where the chloroplast arrangement changed from that of the default geometry (Fig. 2B). The aggregative chloroplast arrangement had a more significant change in the profile of light absorbed across the leaf, which resulted in a peak of absorption around the bundle sheath (Fig. 2B), but also boosted the fractional photon absorption by bundle sheath chloroplasts (Fig. 2C). Although the fractional photon absorption by bundle sheath increased in the avoidance scenario at lower leaf deep, it decreased deeper in the leaf (Fig. 2C).

### Aggregative movement increased ATP and NADPH production in bundle sheath at high light intensities

The effect of chloroplast arrangement on ATP and NADPH production required to balance cell-type-specific demand at limiting light conditions is shown in Table 3. For the default geometry, the absorbed light was mainly used to drive linear electron transport (LET) in mesophyll chloroplasts and cyclic electron transport (CET) in bundle sheath cells. About 40% of the total ATP production was due to bundle sheath chloroplasts. The low NADPH production in bundle sheath cells was due to the assumption of little PSII in bundle sheath cells in the NADP-ME species.

In the aggregative movement case, nearly all of the light absorbed by mesophyll chloroplasts should be used for LET, as in the default case (Table 3). However, ca. 18% more light was allocated to LET in bundle sheath chloroplasts compared with the default. At the bundle sheath cell level, the contribution of CET to the total electron transport rate was only slightly lower in the aggregative than in the default arrangement (Table 3). The aggregative arrangement required ca. 36% more ATP production and ca. 64% more NADPH production in bundle sheath cells.

For avoidance movement, there was increased use of absorbed light by mesophyll and bundle sheath chloroplasts for LET. The increased use of light for LET was ca. 8% more in mesophyll chloroplasts and ca. 11% more in bundle sheath chloroplasts. In addition, the fractions of ATP and NADPH production at limited light intensity (Table 3) increased by ca. 43% and ca. 26% respectively, compared with the default chloroplast arrangement.

The effect of chloroplast arrangement on ATP production was extended to high light intensities where the maximum electron transport rate could be achieved (Fig. 2D). The mean ATP production at high *I*_inc_ was ca. 14–16% higher for aggregative movement compared with the default configuration. The avoidance movement, however, had little effect at *I*_inc_ of 1500 µmol m^−2^ s^−1^ and above. At low *I*_inc_, the avoidance movement decreased the mean ATP production by ca. 15% (Fig. 2D). At limiting *I*_inc_, the total ATP production decreased (Fig. 2D, inset) for all chloroplast movement cases.

### Aggregative chloroplast movement increased *A*_n_ at high light but also increased leakiness

**Fig. 4.**
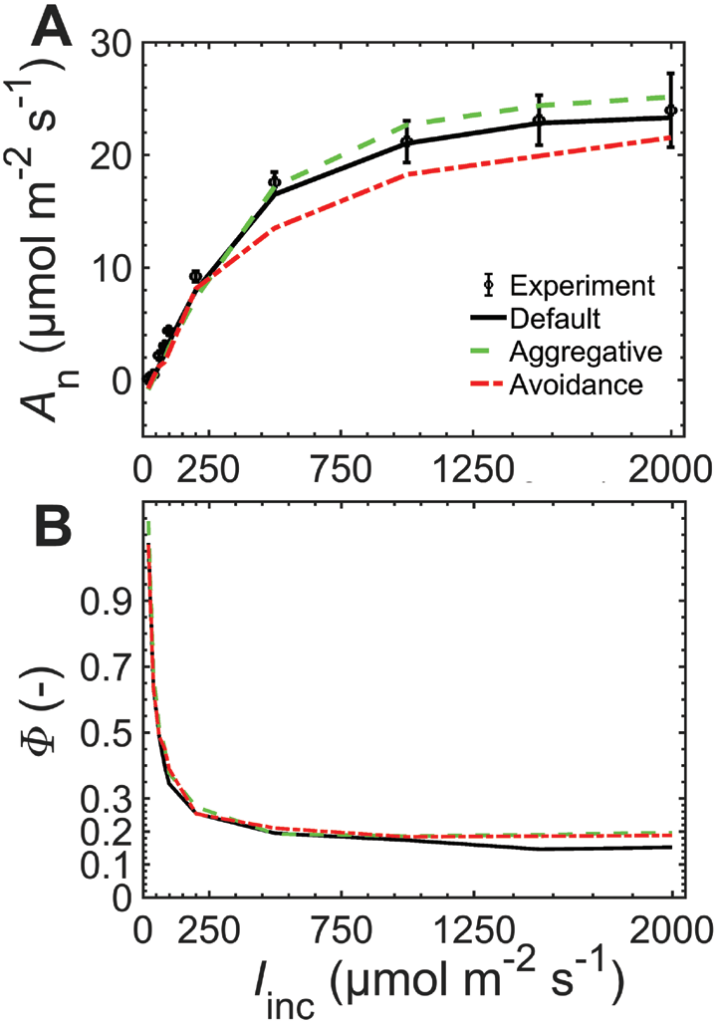
The response of net photosynthesis (*A*_n_) (A) and leakiness (Φ) (B) to changes in irradiance (*I*_inc_). The responses are for default geometry (black solid line), aggregative movement of mesophyll chloroplasts (green dashed line), and the avoidance movement of mesophyll chloroplasts (red dash-dotted line). The responses were computed at ambient CO_2_ of 250 µmol mol^−1^ and 210 mmol mol^−1^ O_2_. Symbols (with error bars) in (A) are for measurement data and lines are for model prediction.

Figure 4A shows the effect of chloroplast arrangement on light response of *A*_n_. Because of the higher ATP production (Fig. 2D), aggregative movement resulted in 4–8% higher *A*_n_ at high *I*_inc_ where the movement may occur. By contrast, *A*_n_ decreased by ca. 12–16% for avoidance movement of mesophyll chloroplasts at *I*_inc_ of 500 µmol m^−2^ s^−1^ and higher. At *I*_inc_ of 1500 µmol m^−2^ s^−1^, Φ was ca. 30% higher for the aggregative and avoidance arrangements than that for the default geometry (Fig. 4B). The increase in Φ for aggregative movement was due to higher CO_2_ in bundle sheath cells and leakage created by the imbalance in CO_2_ assimilation in mesophyll and bundle sheath (Supplementary Fig. S5). The higher ATP production at high *I*_inc_ translated into increased PEP carboxylation as the latter was limited by PEP regeneration, which consumes ATP. The higher PEP carboxylation increased the CO_2_ supply into bundle sheath chloroplasts, where the local Rubisco carboxylation was not proportionally increased (Supplementary Fig. S5). In addition, aggregative and avoidance movements increased oxygen production in bundle sheath cells because of the higher LET (Table 1) thereby increasing photorespiratory CO_2_ release in bundle sheath cytosol (not shown) despite a lower PSII proportion in bundle sheath chloroplasts of maize (assumed here to be 10%).

## Discussion

### Overall performances of the 3D reaction–diffusion model for C_4_ photosynthesis applied to a maize leaf

We developed and tested for the first time a model that concurrently simulates CO_2_ and O_2_ transport, light propagation, C_4_ energetics and related photosynthetic processes in the full 3D geometry of a leaf of the C_4_ model crop maize. The 3D model was primarily validated by comparing simulated and measured responses of photosynthesis to CO_2_ concentration and light intensity. A large part of the merit in this rests on the fine tuning of the 3D light propagation model within a real maize leaf that properly reproduced the *in vivo* absorptance and transmittance measurements. This first 3D model of C_4_ photosynthesis constitutes an advance on the understanding of how anatomy regulates photosynthesis in C_4_ leaves, a complex process that requires fine tuning between the CCM and light available for chloroplasts in mesophyll and bundle sheath cells. The anatomical features influencing gas diffusion and light propagation compared well with literature values, although the features vary with treatments (Table 1) (Dengler *et al.*, 1994; von Caemmerer *et al*., 2007; Retta *et al.*, 2016b). The 3D model was applied in analysing photosynthesis by addressing the role of chloroplast arrangement, which requires a representative tissue-level 3D geometry and could not be realistically carried out by a 2D model (Supplementary Fig. S4) (Théroux-Rancourt *et al.*, 2017; Earles *et al.*, 2019). The 3D light profile showed that large photon absorption gradients existed when the Kranz anatomy was illuminated unilaterally depending on the 3D chloroplast distribution across the leaf thickness. The large gradient means that some of the chloroplasts are still light limited (Figs 1D, 2B; 18% by volume of the chloroplast absorbed less than 0.001% of the photons) and may strongly contribute to the non-saturated irradiance response of the leaf-level photosynthesis. However, light may be scattered more than modeled here by cell walls (Gates *et al.*, 1965; Allen *et al.*, 1973; Vogelmann, 1993), which were not explicitly included in the geometry because of the requirement of a much denser mesh and, hence, computational demand, but the scattering effect was lumped with the chloroplasts. Light may also penetrate deeper in leaf tissue in C_4_ leaves having bundle sheath extensions (Bellasio and Lundgren, 2016), which were not included here because bundle sheath extensions were not observed in the maize leaf anatomy (Supplementary Fig. S1A). In addition, the 3D model could be validated more accurately by comparing the intra-leaf profile of light and photosynthetic capacity. For instance, light-sheet microscopy can be used to resolve the profile of light across the leaf thickness (Slattery *et al.*, 2016). In addition, the profile of photosynthetic capacity could also be estimated using epi-illumination fluorescence microscopy to derive the profile of chlorophyll and Rubisco, together with the aforementioned light absorption profile (Vogelmann and Evans, 2002; Slattery *et al.*, 2016; Borsuk and Brodersen, 2019). Overall, the heterogeneity in light and [CO_2_] suggests that the contributions of electron transport and enzyme-limited CO_2_ assimilation rates to the total assimilation are influenced by the leaf microstructure.

### Effect of mesophyll chloroplast movement on CO_2_ concentrating mechanism efficiency and photosynthetic rate

The 3D gas transport model was coupled with the ATP and NADPH production model using the validated light propagation model and was applied to test hypotheses on the effect of chloroplast movement on CCM efficiency and photosynthetic rate. The default 3D geometry was modified to model the geometries of the various arrangements of mesophyll and bundle sheath chloroplast scenarios inspired by what is observed *in vivo* from microscopy imaging (Yamada *et al.*, 2009; Maai *et al.*, 2011, 2020a,b; Sales *et al.*, 2018). Care was taken not to modify the chloroplast volume fraction at the cell level in addition to the total chloroplast volume fraction so that the absorption and scattering coefficients remained similar. Consistent with previous observations, chloroplast movement increased transmittance and decreased light absorption (Yamada *et al.*, 2009; Sun *et al.*, 2014; Maai *et al.*, 2020a). Avoidance movement was slightly more effective in decreasing total light absorption by chloroplasts. By contrast, aggregative movement highly increased absorption by bundle sheath chloroplasts while reducing the light exposure of mesophyll chloroplasts (Table 3). Although the aim of this study was to test the efficiency of mesophyll chloroplasts in gas exchange rates, our findings of light absorption profiles could be used in further studies to evaluate mechanisms of photoprotection and photoinhibition in C_4_ leaves when abiotic stress results in chloroplast movement (Ghannoum, 2009; Guidi *et al.*, 2019).

The 3D model predicts that chloroplast movement impacted photosynthetic rate and CCM efficiency through altered light availability in mesophyll and bundle sheath chloroplasts, energy production, and changes in the process of electron transport. At high light intensities, where the aggregative movement was expected, there was a strong increase in photosynthetic rate (Fig. 4A) supporting our hypothesis that aggregative movement of mesophyll chloroplasts improves photosynthetic rate. In addition, avoidance movement of mesophyll chloroplasts decreased ATP production and *A*_n_ and increased Φ at high light intensities compared with the default case of no chloroplast movement. Thus, the hypothesis that avoidance movement decreases photosynthetic rate was supported. These results support the previous suggestion that preference for aggregative movement rather than avoidance movement in maize upon exposure to blue light intensity may have benefits for photosynthesis (Ryu *et al.*, 2014). The altered light absorbance by mesophyll and bundle sheath chloroplasts created an imbalance in the rate of CO_2_ assimilation in mesophyll and bundle sheath cells resulting in increased leakiness (Fig. 4b). Such an imbalance has recently been shown to increase leakiness due to lack of coordination between mesophyll and bundle sheath cells following dark to high light transition (Wang *et al.*, 2022). Previous modeling results suggested that increased leakiness may be beneficial as it helps balance reducing power demand and availability by removing excess CO_2_ from bundle sheath cells (Wang et al., 2014; Kromdijk *et al.*, 2014). Balancing ATP and NADPH demand of the CCM required an increased operation of linear electron transport in mesophyll cells due to less light absorption by mesophyll chloroplasts (Table 3). Overall, the results support the hypothesis that chloroplast movement, although useful for photoprotection because less light is absorbed by chloroplasts, may affect the operation of CCM and reduce its efficiency (Taniguchi *et al.*, 2003; Yamada *et al.*, 2009; Maai *et al.*, 2011, 2020b; Sun *et al.*, 2014). These results are clear indications that C_4_ photosynthesis can be improved by optimizing light absorption and increasing ATP and NADPH production (Ermakova *et al*., 2019, 2021b), but at the expense of CCM efficiency. These results are relevant for stacking traits in the C_4_ Rice project (Ermakova *et al.*, 2021a; Xiao *et al.*, 2023), for example, one should consider designing strategies of boosting Rubisco activity in order to remedy the lower CCM efficiency in addition to reducing the limitation by electron transport at high light intensity by boosting electron transport capacity in C_4_ plants (Ermakova *et al*., 2019).

Chloroplast movement in C_4_ plants, unlike in C_3_ plants, requires exposure to high light intensity for several hours, which can be experienced by leaves close to the top of the canopy. The long duration may have been due to the sparse arrangement of chloroplasts in mesophyll cells of C_4_ plants making them less susceptible (Ghannoum *et al.*, 2005; Stata *et al.*, 2014), an evolutionary adaption of C_4_ plants, which may allow more light to pass through to bundle sheath cells (Stata *et al.*, 2014). Our results suggest that CCM efficiency may also add another constraint, which needs to be verified experimentally. In addition, the limitation of metabolite transport may explain the limited movement of maize bundle sheath chloroplasts (Taniguchi *et al.*, 2003; Kato *et al.*, 2022). Our simulation results suggest that aggregative movement may provide benefits for improved photosynthesis besides photoprotection. However, if mesophyll chloroplasts are not arranged in this mode most of the day, it means that random distribution of chloroplasts in mesophyll cells is needed for processes other than photosynthesis, and only when light intensity is really high does the advantage of this configuration for gas exchange and photoprotection overcome the benefits of the other chloroplast functions within mesophyll cells.

### Concluding remarks

We developed a 3D R-D model of gas exchange and light propagation coupled to an ATP and NADPH production model and applied it to test the hypotheses that movement of chloroplasts reduces light absorption within the C_4_ leaf but modifies differently the CCM efficiency and photosynthesis depending on the type of mesophyll chloroplast movement. We found that mesophyll aggregative chloroplasts arrangement increased the proportion of light absorption by bundle sheath cells, the leaf ATP and NADPH production, and photosynthetic rate. By contrast, the avoidance arrangement decreased photosynthesis. None of the chloroplast movement scenarios were beneficial for maintaining CCM efficiency. C_4_ plants could boost photosynthetic rate further at the expense of increased leakiness using chloroplast movement in addition to photo-protection under high light intensity (Yamada *et al.*, 2009; Maai *et al.*, 2011, 2020b). Furthermore, evidence has been provided that a 3D model, unlike simpler biochemical models and a 2D model, can provide increased mechanistic understanding of the light propagation, CO_2_ diffusion, and chloroplast movement in complex C_4_ photosynthesis.

## Supplementary data

The following supplementary data are available at *JXB* online.

Fig. S1. Sample light microscopy images of maize leaf tissue.

Fig. S2. Stomatal conductance to CO_2_ in response to irradiance and degree of stomatal opening.

Fig. S3. Comparison of volume of mesophyll chloroplasts per cell.

Fig. S4. Comparison of a 2D and a 3D model.

Fig. S5. Comparison of local rates and CO_2_ concentration.

Table S1. List of symbols, their definitions, and units.

Table S2. Mean equivalent radius and total number of organelles per volume.

Table S3. Computed optical properties of the different compartments of the leaf model.

erad138_suppl_Supplementary_materials

## Data Availability

The MATLAB code used for solving the reaction–diffusion models is available at Dryad Digital Repository at https://doi.org/10.5061/dryad.59zw3r2bx (Retta *et al*., 2023).
