# Supplementary material for: The role of chloroplast movement in C4 photosynthesis: a theoretical analysis using a three-dimensional reaction–diffusion model for maize
Source: J Exp Bot. 2023 Apr 21;74(14):4125–42. doi: 10.1093/jxb/erad138 (PMC10400148; doi:10.1093/jxb/erad138)
Supplement: erad138_suppl_Supplementary_materials [file erad138_suppl_supplementary_materials.pdf]

The role of chloroplast movement in C_4 photosynthesis: A theoretical analysis using a 3-D reaction-diffusion model for maize

M.A. Retta[1,2], X. Yin[2], Q.T. Ho[3], R. Watté[1], H.N.C. Berghuijs[4], P. Verboven[1], W. Saeys[1], F.J. Cano[5,6], O. Ghannoum[6], P.C. Struik[2,*], B.M. Nicolaï[1,7,*]

[1] KU Leuven, MeBioS division, Willem de Croylaan 42, B-3001, Leuven, Belgium

[2] Centre for Crop Systems Analysis, Wageningen University & Research, P.O. Box 430, 6700 AK Wageningen, The Netherlands

[3] Institute of Marine Research, Nordnesgaten 50, NO-5005 Bergen, P.O. Box 1870, Nordnes, Norway

[4] Plant Production Systems group, Wageningen University & Research, P.O. Box 430, 6700 AK Wageningen, The Netherlands

[5] Centro de Investigación Forestal (CIFOR), Instituto Nacional de Investigacion y Tecnologia Agraria y Alimentaria (INIA), Consejo Superior de Investigaciones Científicas (CSIC), Carretera de la Coruña Km 7.5, 28040, Madrid, Spain

[6] ARC Centre of Excellence for Translational Photosynthesis, Hawkesbury Institute for the Environment, University of Western Sydney, Hawkesbury campus, Locked Bag 1797, Penrith 2751, NSW, Australia

[7] Flanders Center of Postharvest Technology, Willem de Croylaan 42, B-3001, Leuven, Belgium

* Corresponding authors:

Bart M. Nicolaï, Flanders Center of Postharvest Technology / BIOSYST-MeBioS, KU Leuven, Willem de Croylaan 42, B-3001 Leuven, Belgium

Email: bart.nicolai@kuleuven.be Tel: +32 16 322375 Fax: +32 16 322955

Paul C. Struik, Centre for Crop Systems Analysis, Wageningen University & Research, P.O. Box 430, 6700 AK Wageningen, The Netherlands

Email: paul.struik@wur.nl Tel: + 31 317 484246

Figures

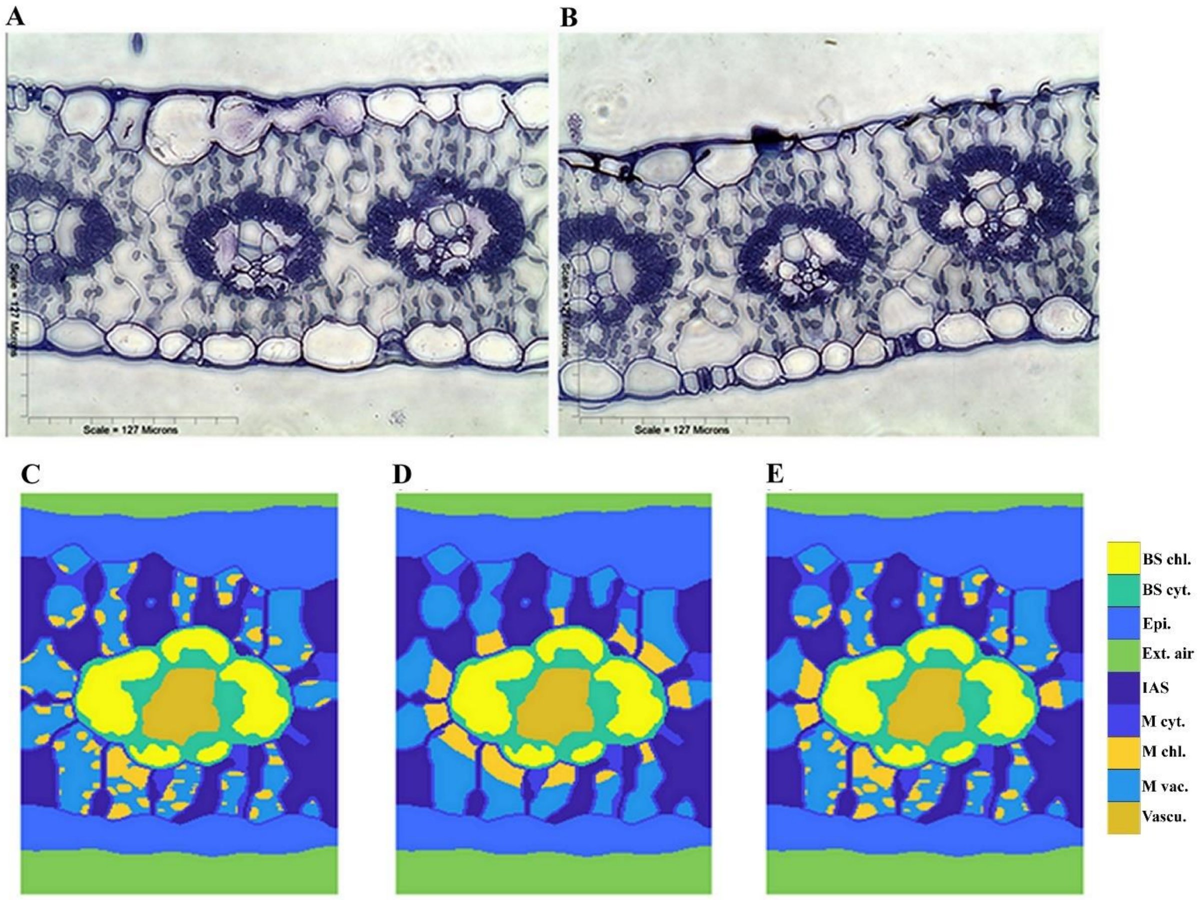

Figure S1. Sample light microscopy images of maize leaf tissue. Shown in (A) is 48th section while in (B) is 91th in the stack of 120 semi-thin sections. A slice (slice 102) from the default geometry (C), aggregative movement of mesophyll chloroplasts (D) and avoidance movement (E) are shown. Color bar show chloroplast (chl.), cytosol (cyt.) of bundle-sheath (BS) or mesophyll (M) cells, epidermis (Epi.), domain of external air (Ext. air), intercellular air space (IAS), vacuole (Vac.) and vascular bundle (Vascu.).

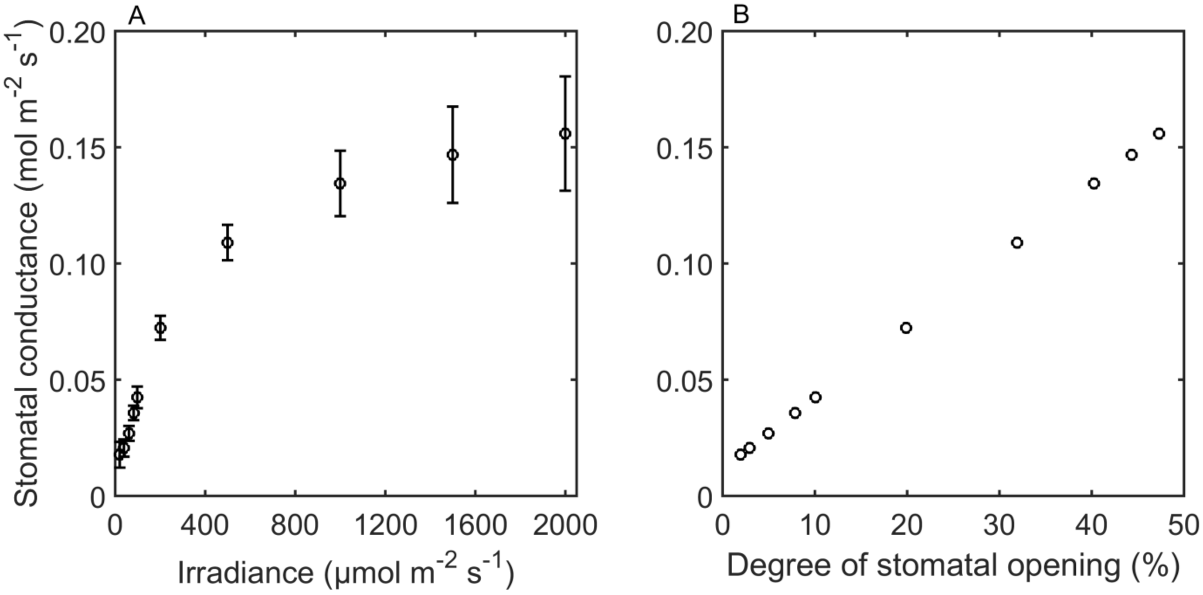

Figure S2. Stomatal conductance to CO_2 in response to irradiance and degree of stomatal opening. Response of stomatal conductance to irradiance (A) measured at ambient CO_2 of 250 µmol mol^{-1} and 210 mmol mol^{-1} O_2, leaf temperature of 25 °C and leaf-to-air vapor pressure difference within 1.0−1.6 kPa. The stomatal conductance was modeled by adjusting the diffusion coefficient of gases in the stomata to simulate various degrees of stomatal opening (B). Error bars represent standard error (n = 4).

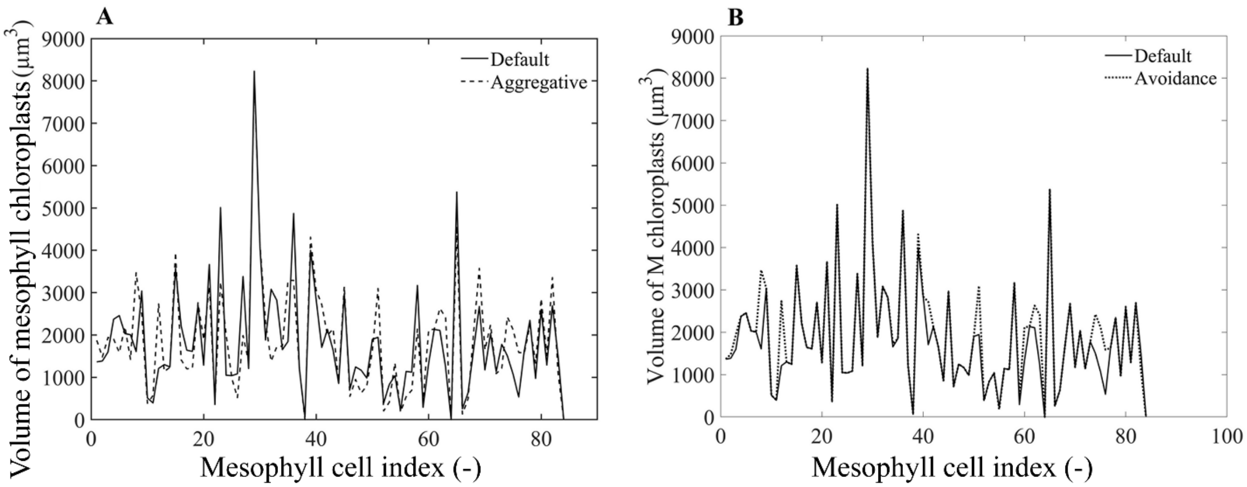

Figure S3. Comparison of volume of mesophyll chloroplasts per cell for default (solid line), aggregative movement (dashed) and avoidance (dotted line). Panel A shows a comparison between default and aggregative and Panel B shows that of default and avoidance.

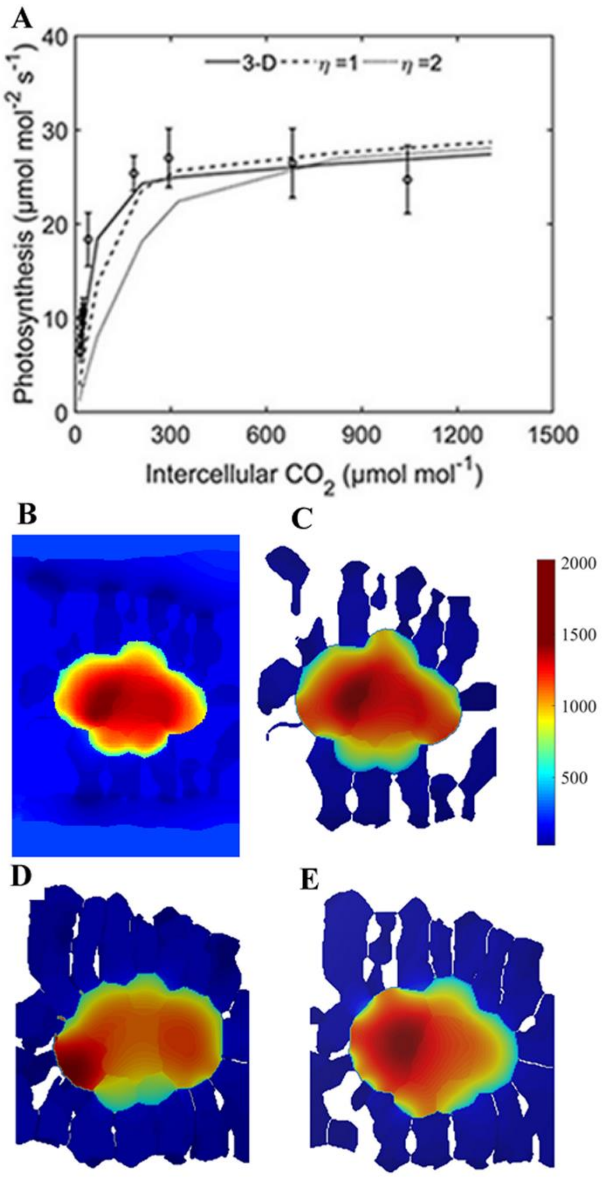

Figure S4. Comparison of a 2-D and a 3-D model. The response of photosynthesis to intercellular CO_2 (A) predicted from the 2-D model for two values of the relative viscosity (viscosity of cytosol divided by viscosity of water) of mesophyll and bundle sheath cytosol (η): η = 1 (dashed line), η = 2 (dotted line) and from the 3-D model using η = 2 (solid line). Symbols represent experimentally measured values (n = 4). CO_2 concentration profiles within a maize leaf obtained from a 3-D model (η = 2), slice 60 (B), a profile from a 2-D model (η = 2) solved on slice 60 (C), slice 30 (D) and slice 90 (E) assuming the intercellular air space is fully connected. The color bar is concentration of CO_2 in μmol mol^{-1}.

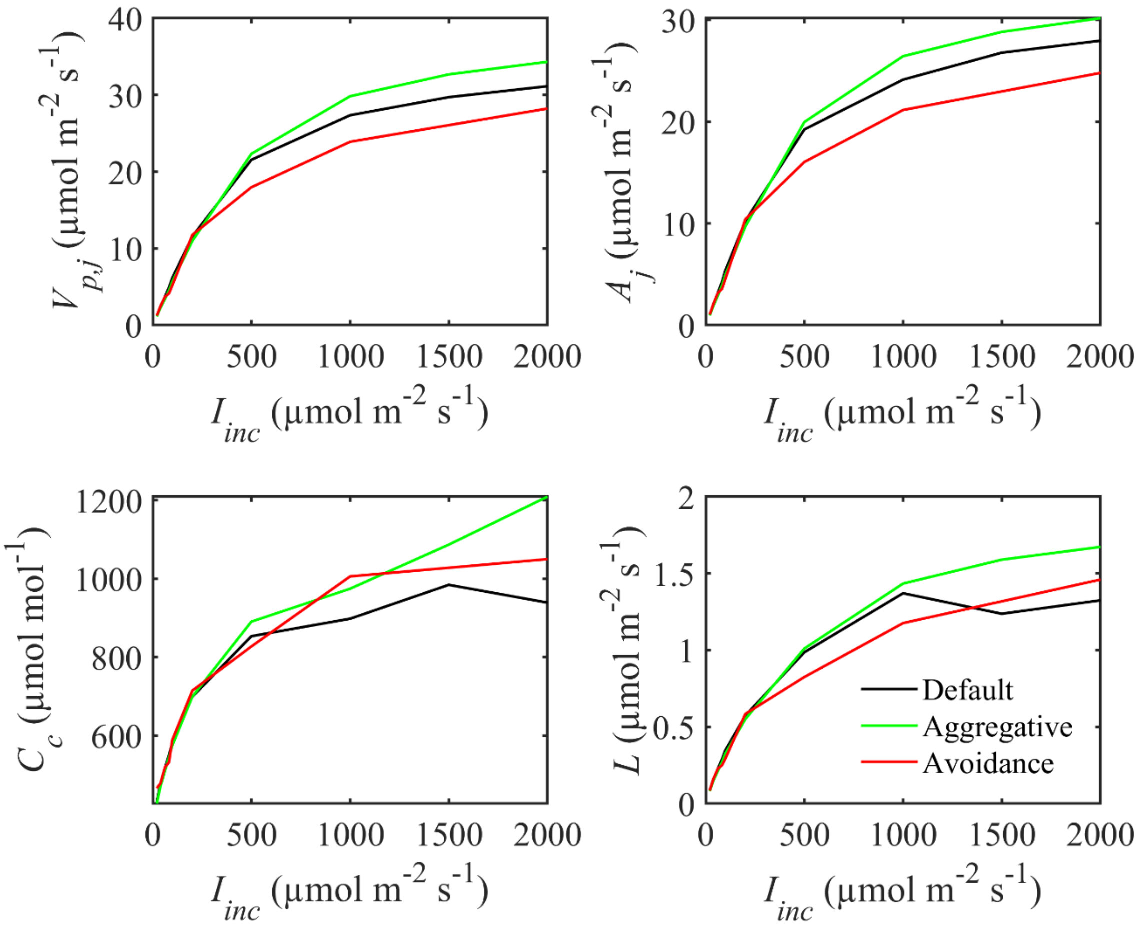

Figure S5. Comparison of local rates and CO_2 concentration. The response to irradiance (I_{inc}) of PEP carboxylation rate limited by ATP ($V_{p,j}$) and ATP limited rate of photosynthesis ($A_{j,ATP}$), CO_2 concentration in bundle-sheath cells (C_c) and leakage rate (L) for default geometry (black line), aggregative (green line) and avoidance movement (red line).

Tables

Table S1. List of symbols, their definitions and units

Variable	Definition
A_{n}	Volumetric rate of net photosynthesis (μmol m^{-3} s^{-1})
A_{n}^{*}	Volumetric rate of net photosynthesis (μmol m^{-3} s^{-1})
B_{CA}	Net hydration of CO_2 in presence of carbonic anhydrase (μmol m^{-3} s^{-1})
B_{NCA}	Non-enzymatic net hydration of CO_2 (μmol m^{-3} s^{-1})
d	Average thickness of leaf tissue (μm)
$E_{\mathrm{O_2}}^{*}$	Volumetric rate of oxygen evolution (μmol m^{-3} s^{-1})
$f_{\mathrm{ATP,BS}}$	Fraction of ATP produced in bundle-sheath (BS) chloroplasts
f_i	Volume fraction of a cell component *i* in the leaf tissue (chloroplasts, cytosol, epidermis, vascular bundles)
$f_{\mathrm{CET,BS}}$	Fraction of cyclic e$^-$ transport (CET) in BS chloroplasts
$f_{\mathrm{NADPH,BS}}$	The fraction NADPH in bundle-sheath (BS) chloroplast
f_{resp}	Total volume fraction of respiratory tissue in the leaf
$I_{\mathrm{abs},i}^{*}$	Rate of photon absorption by bundle-sheath or mesophyll chloroplasts (subscript *i*) per unit leaf volume (μmol m^{-3} s^{-1})
j^{*}	Potential rate of e$^-$ transport per unit leaf volume (μmol m^{-3} s^{-1})
j_{ATP}	Total rate of ATP production in the leaf (μmol m^{-2} s^{-1})
j_{ATP}^{*}	Total rate of ATP production per unit leaf volume (μmol m^{-3} s^{-1})
$j_{\mathrm{ATP},i}$	Total rate of ATP production in mesophyll or bundle-sheath chloroplasts (subscript *i*) (μmol m^{-2} s^{-1})
$j_{\mathrm{ATP},CF}$	Total rate of ATP production calculated from chlorophyll fluorescence (CF) measurement (μmol m^{-2} s^{-1})
$j_{\mathrm{NADPH},i}$	Total NADPH produced in mesophyll or bundle-sheath cells (subscript *i*) (μmol m^{-2} s^{-1})
j_{ATP}^{*}	Total rate of ATP production per unit leaf volume (μmol m^{-3} s^{-1})
j_{LL}^{*}	Potential rate of light-limited (LL) e$^-$ transport per unit leaf volume (μmol m^{-3} s^{-1})
$j_{\mathrm{LL,CET},i}^{*}$	Potential rate of light-limited (LL) cyclic e$^-$ transport (CET) in bundle-sheath (BS) or mesophyll (M) cells per unit leaf volume (subscript *i*) (μmol m^{-3} s^{-1})
$j_{\mathrm{LL,LET},i}^{*}$	Potential rate of light-limited (LL) linear e$^-$ transport (LET) in bundle-sheath (BS) or mesophyll (M) cells per unit leaf volume (subscript *i*) (μmol m^{-3} s^{-1})
j_{max}^{*}	Rate of light-saturated e$^-$ transport per unit leaf volume (μmol m^{-3} s^{-1})
L	Rate of CO_2 leakage (μmol m^{-2} s^{-1})

R^*	Volumetric rate of CO_2 release through respiration in M and BS cells, epidermis and vascular bundles (μmol m^{-3} s^{-1})
R^*_{EPI}	Rate of respiratory CO_2 release from epidermis cells per unit leaf volume (μmol m^{-3} s^{-1})
R^*_i	Rate of respiratory CO_2 release from cell component *i* (mesophyll cytosol, bundle-sheath cytosol or vascular bundles) per unit leaf volume (μmol m^{-3} s^{-1})
r^*	Total O_2 consumption due to RuBP oxygenation during photorespiration (μmol m^{-3} s^{-1})
r^*_p	Rate of photorespiratory CO_2 release in bundle-sheath cytosol per unit leaf volume (μmol m^{-3} s^{-1})
r^*_{p,O_2}	Rate of O_2 consumption due to photorespiratory in bundle-sheath chloroplasts per unit leaf volume (μmol m^{-3} s^{-1})
S_{leaf}	Area of a cross section of the computational domain (m^2)
u_M	Fraction of light for LET in mesophyll (M) chloroplasts
u_{BS}	Fraction of light for LET in bundle-sheath (BS) chloroplasts
$V^*_{c,max}$	Maximum rate of Rubisco activity-limited carboxylation per unit leaf volume (μmol m^{-3} s^{-1})
$V^*_{CA,max}$	Maximum catalytic activity of CA per unit leaf volume (μmol m^{-3} s^{-1})
V_p	Rate of PEP carboxylation based on bicarbonate ions (μmol m^{-2} s^{-1})
V^*_p	Rate of V_p per unit leaf volume (μmol m^{-3} s^{-1})
$\overline{V_p}$	Average rate of CO_2 production through decarboxylation of C_4 acids in the BS chloroplasts per unit leaf area (μmol m^{-2} s^{-1})
$\overline{V^*_p}$	Average rate of CO_2 production through decarboxylation of C_4 acids in the BS chloroplasts per unit leaf volume (μmol m^{-3} s^{-1})
w^*_c	Volumetric rate of Rubisco-limited carboxylation (μmol m^{-3} s^{-1})
$w^*_{j,ATP}$	Volumetric rate of Electron-transport-limited carboxylation (μmol m^{-3} s^{-1})
$\alpha_{2,LL}$	Photochemical efficiency of linear e^- transport of PSII under limiting light (LL) when CET occurs simultaneously (based on light absorbed by both photosystems)
Φ	Leakiness

Table S2. Mean equivalent radius and total number of organelles per volume in mesophyll (M) and bundle-sheath (BS) cells used for modeling light absorption.

Organelle type	Mean equivalent radius $\bar{r}$ (µm) [(a)]	f_{tot} [(b)] (number m^{-3})	References
Mitochondria			(Yoshimura *et al.*, 2004; Dieteren *et al.*, 2011)
M cells	0.04	0.020	
BS cells	0.07	0.0096	
Peroxisomes	0.25 ± 0.0025	0.00273	(Yoshimura *et al.*, 2004).
Nuclei	2.57 ± 0.0899	0.000201	(Dittmer *et al.*, 2007)
Golgi stacks	0.42 ± 0.0917	0.002518	(Dupree and Sherrier, 1998)
Ribosome-like complexes	0.0137 ± 0.000625	445.44	(Verschoor *et al.*, 1998)
Grana	0.472 ± 0.0558	0.68	(Staehelin, 2003; Yoshimura *et al.*, 2004; Vicankova and Kutik, 2005; Austin and Staehelin, 2011)

[(a)] The equivalent radius of an organelle having volume $\overline{V}_{organelle}$ is given by $\bar{r} = \left(\frac{3\overline{V}_{organelle}}{4\pi}\right)^{1/3}$

The lowest and highest value obtained from literature were used to determine the standard deviation.

[(b)] f_{tot} is the product of the number of organelles per unit volume.

Table S3. Computed optical properties of the different compartments of the leaf model. The absorption profile is the result of averaging the absorption profile at 470 nm (10 %) and at 665 nm (90 %). μ_a is an absorption coefficient; μ_s is a scattering coefficient; and, γ is an anisotropy factor.

470 nm	$\mu_a\ (cm^{-1})$	$\mu_s\ (cm^{-1})$	$\gamma\ (-)$
Air	0	2000	1
Epidermis	10	730	0.9678
Mesophyll cytosol	10	850	0.9800
Mesophyll vacuole	10	730	0.9556
Mesophyll chloroplast	3800	12700	0.9698
Bundle-sheath cytosol	10	730	0.9678
Bundle-sheath chloroplast	1900	12700	0.9678
Vascular bundles	10	1000	0.9800
665 nm			
Air	0	2000	1
Epidermis	10	330	0.9400
Mesophyll cytosol	10	450	0.9600
Mesophyll vacuole	10	330	0.9200
Mesophyll chloroplast	3400	10700	0.9698
Bundle-sheath cytosol	10	330	0.9500
Bundle-sheath chloroplast	1700	10700	0.9678
Vascular bundles	10	500	0.9600

References

Austin JR, Staehelin LA. 2011. Three-dimensional architecture of grana and stroma thylakoids of higher plants as determined by electron tomography. Plant physiology **155**, 1601–11.

Dieteren CEJ, Gielen SCAM, Nijtmans LGJ, Smeitink JAM, Swarts HG, Brock R, Willems PHGM, Koopman WJH. 2011. Solute diffusion is hindered in the mitochondrial matrix. Proceedings of the National Academy of Sciences of the United States of America **108**, 8657–62.

Dittmer TA, Stacey NJ, Sugimoto-Shirasu K, Richards EJ. 2007. Little nucleic genes affecting nuclear morphology in Arabidopsis thaliana. The Plant cell **19**, 2793–803.

Dupree P, Sherrier DJ. 1998. The plant Golgi apparatus. Biochimica et biophysica acta **1404**, 259–70.

Staehelin LA. 2003. Chloroplast structure: from chlorophyll granules to supra-molecular architecture of thylakoid membranes. Photosynthesis research **76**, 185–96.

Verschoor A, Warner JR, Srivastava S, Grassucci R a, Frank J. 1998. Three-dimensional structure of the yeast ribosome. Nucleic acids research **26**, 655–61.

Vicankova A, Kutik J. 2005. Chloroplast ultrastructural development in vascular bundle sheath cells of two different maize (Zea mays L.) genotypes. Plant soil and environment **51**, 491–495.

Yoshimura Y, Kubota F, Ueno O. 2004. Structural and biochemical bases of photorespiration in C4 plants: quantification of organelles and glycine decarboxylase. Planta **220**, 307–317.
